# Supramolecular nanofibers of natural asiaticoside for self-supporting gelation and enhanced transdermal delivery

**DOI:** 10.3389/fbioe.2025.1589865

**Published:** 2025-05-02

**Authors:** Xixi Hu, Shuang Tian, Jiao Wang, Weixi Luo, Jiangli Yao, Rui Zhu, Yiyuan Dai, Hongyun Li, Yuhua Ma, Chen Liu, Wenping Wang

**Affiliations:** ^1^ College of Chinese Materia Medica, Yunnan University of Chinese Medicine, Kunming, Yunnan, China; ^2^ Key Laboratory for Tibet Plateau Phytochemistry of Qinghai Province, School of Pharmacy, Qinghai Nationalities University, Xining, Qinghai, China; ^3^ General Hospital of Ningxia Medical University, Yinchuan, Ningxia, China

**Keywords:** asiaticoside, supramolecular hydrogel, self-assembled nanofibers, transdermal drug delivery, natural product gelator, molecular dynamics simulation

## Abstract

**Objectives:**

The study aimed to develop a supramolecular hydrogel of asiaticoside (AS) via self-assembly and evaluate its potential for enhanced transdermal delivery.

**Materials and Methods:**

AS was dissolved in dimethyl sulfoxide (DMSO) and dispersed into a glycerol–water mixture (3:7 v/v) via ultrasonication to induce gelation. The critical gelation concentration (CGC) was determined through macroscopic and microscopic evaluation. Morphological analysis was performed using various microscopy techniques. Physicochemical properties were assessed using differential scanning calorimetry (DSC), powder X-ray diffraction (PXRD), Fourier-transform infrared (FTIR) spectroscopy, and UV–VIS spectroscopy. Molecular dynamics (MD) simulations with general AMBER force field (GAFF) parameters were used to analyze assembly dynamics. Rheological behavior and transdermal performance were tested using a rheometer and Franz diffusion cells, respectively.

**Results:**

The hydrogel formed at a CGC of 0.5% w/v, exhibiting pH-responsive gelation and a nanofibrous architecture. MD simulations revealed hydrogen bonding and π–π stacking as the dominant drivers of assembly, supported by FTIR peak shifts. The hydrogel demonstrated shear-thinning behavior (G’ > G″) and thermal stability below 70°C. Compared to the AS suspension, the hydrogel enhanced transdermal flux by 1.73-fold and skin retention by 2.04-fold, attributed to supersaturated drug molecules and sustained release from the nanofiber network.

**Conclusion:**

This work pioneers the use of AS as a natural supramolecular gelator, addressing its bioavailability challenges through nanostructured self-assembly. The hydrogel’s dual functionality (pH-responsive gelation and enhanced permeation) offers a sustainable platform for the transdermal delivery of hydrophobic phytochemicals, bridging phytochemistry and nanobiotechnology. This strategy expands the application of plant-derived saponins in advanced drug delivery systems.

## 1 Introduction

Asiaticoside (AS), a triterpenoid saponin isolated from *Centella asiatica*, has gained recognition for its dermatological applications due to its anti-inflammatory, antioxidant, and wound-healing properties (Bandopadhyay et al., 2023; Bylka et al., 2013; He et al., 2023). Its ability to stimulate collagen synthesis makes it valuable in cosmetic formulations and scar management therapies (Bylka et al., 2014). However, the clinical utilization of AS is constrained by limited aqueous solubility (0.7 mM), poor lipophilicity, and low skin-permeation efficiency, which are attributed to its high molecular weight (959.12 g/mol) and amphiphilic structure (Hengjumrut et al., 2018; Soe et al., 2020). To address these challenges, formulation strategies such as nanosuspensions, nanoemulsions, and hydrogel-based systems have been explored (Kim et al., 2021; Li et al., 2020; Wichayapreechar et al., 2020; Witkowska et al., 2023).

**FIGURE 1:**
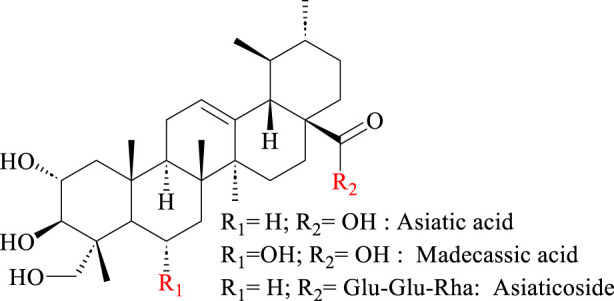
Chemical structure of asiaticoside, asiatic acid, and madecassic acid.

Hydrogels, with their tunable rheology and biocompatibility, are particularly promising for topical drug delivery (Fan et al., 2021; Labie and Blanzat, 2023; Pourtalebi Jahromi et al., 2023). Recent advancements highlight the potential of natural product-based hydrogels, which combine stimuli-responsiveness with inherent bioactivity (Feng et al., 2021). Triterpenoids, known for their self-assembly capabilities via non-covalent interactions, have been engineered into micelles and nanocarriers (Böttcher and Drusch, 2017; Lu and Ju, 2016; Malík et al., 2021). Although asiatic acid and madecassic acid (Figure 1) exhibit micelle-forming behavior in aqueous solutions (Rafat et al., 2008; Stephenson et al., 2008), the self-assembly potential of AS remains unexplored despite its structural similarity to these compounds.

This study investigates the supramolecular gelation behavior of AS in aqueous media. We systematically characterize the self-assembly process, hydrogel properties, and underlying molecular interactions through experimental and computational approaches. Additionally, we evaluate the hydrogel’s skin permeation performance as a foundational step toward developing natural product-based transdermal delivery systems.

## 2 Materials and methods

### 2.1 Materials

Asiaticoside (HPLC purity >98%) was obtained from Jingzhu Biotechnology Co., Ltd. (Nanjing, China). Dimethyl sulfoxide (DMSO) and analytical-grade solvents were purchased from Zhiyuan Chemical Reagent Co., Ltd. (Tianjin, China). Spectral-grade potassium bromide (KBr) was supplied by Guangfu Technology Development Co., Ltd. (Tianjin, China). Isoflurane was purchased from Shandong Ante Animal Husbandry Technology Co., Ltd. (Shandong, China). Ultra-purified water was generated using an Arium^®^ Comfort II System (Sartorius, Germany).

Kunming mice, weighing 25 ± 10 g, were provided by the Experimental Animal Center of the Yunnan University of Chinese Medicine (SYXK-K2022-0004). All animal studies were carried out in accordance with the guidelines for assessment and approval by the Ethics Committee of Yunnan University of Chinese Medicine.

### 2.2 Gel preparation and characterization

#### 2.2.1 Gelation screening

The potential gelling medium for AS was screened via a simple ultrasonication-aided anti-solvent dispersion method described as follows (Ma et al., 2024): AS (40% w/v in DMSO) was dispersed into aqueous media (pH 2.0–9.2 PBS) or glycerol–water mixtures (3:7 v/v) using a sonicator (40 kHz, Ningbo, China) for 5 min. Gel formation was assessed visually and via optical microscopy (XS-212, Jiangnan, China) at 2% w/v AS concentration.

#### 2.2.2 Critical gelation concentration

The minimum AS concentration (0.5%–2% w/v) required for gelation in glycerol–water systems (3:7 w/w) was determined via the inverted vial method and verified under microscopy. Gelation occurs if no content flows down after inversion.

### 2.3 Morphological observation

#### 2.3.1 Polarized light microscopy

Bulk AS and hydrogels were imaged using an MXP-260 microscope (Nanjing, China) to detect birefringence.

#### 2.3.2 Electron microscopy

Freeze-dried xerogels were sputter-coated with gold and analyzed using a scanning electron microscope (SEM, Phenom Pro, Netherlands). Hydrogel nanostructure was visualized using a transmission electron microscope (TEM, HT7800, Hitachi, Japan) after phosphotungstate staining.

### 2.4 Physicochemical characterization

#### 2.4.1 Thermal analysis

The differential scanning calorimetry (DSC) profiles (DSC 3500 Sirius, NETZSCH, Germany) were recorded from 25°C to 390°C (10°C/min, N_2_ atmosphere).

#### 2.4.2 Crystallinity

The powder X-ray diffractogram (PXRD) patterns (D8 Advance, Bruker, Germany) were acquired at 5°–90° 2*θ* (5°/min).

#### 2.4.3 Molecular interactions

The Fourier-transform infrared (FTIR) spectrum spectra (GREAT20, CKRG, China) and the ultraviolet-visible (UV–VIS) spectroscopy profiles (T6, PGeneral, China) were collected at 400–4,000 cm^-1^ and 200–800 nm, respectively.

### 2.5 Molecular dynamic simulations

Initially, the molecular conformation of AS was optimized via the B97-3c (Brandenburg et al., 2018) method using the ORCA software package (Neese, 2022). In addition, the parameters for the general AMBER force field (GAFF) were generated using Sobtop software (Lu, 2024). MD simulation was performed using the GROMACS 2022.5 program (Van Der Spoel et al., 2005); a box was constructed with 30 AS molecules, 1,000 glycerol molecules, and 11,000 TIP3P water molecules. In the process of MD simulation, the LINCS algorithm was used to constrain all hydrogen bonds involved. The electrostatic interaction was calculated by the particle-mesh Ewald (PME) method, the van der Waals interaction was set to cutoff, and the non-bonded interaction cutoff was set to 10 A. The energy of the system was minimized by the steepest descent method and the conjugated gradient method to eliminate the unreasonable contact between atoms. Then, the NPT equilibrium simulation was performed for 200 ps, the V-rescale temperature coupling method was used to control the simulated temperature to 298.15 K, the Berendsen method was used to control the pressure to 1 bar, and the integration step was 2 fs. The formal simulation was carried out for 100 ns, the pressure method was switched to the Parrinello–Rahman method, and the trajectory was saved every 10 ps. The simulation results were visualized using VMD (Humphrey et al., 1996).

### 2.6 Rheology test

Rheological tests were conducted using an MCR 102e rheometer (Anton Paarl, Austria) equipped with a 25 mm plate–plate. The viscosity of different concentrations of AS gels was measured in the shear rate range of 0.1–10 [1/s]. Oscillatory shear was applied at 1 Hz and 0.01%–100% strain to collect the equilibrium storage (G′) and the loss (G″) moduli for the determination of the linear visco-elastic region. The frequency sweep was performed in a range of 0.1–100 rad/s at 2% strain and 25°C, and the strain amplitude sweep was carried out over a range of 1%–100% strain at 1 Hz and 25°C. Degradation over temperature was assessed by the determination of G in a range of 20°C–80°C at a heating rate of 15°C/min.

### 2.7 Skin permeation test

The 2% AS hydrogel was evaluated for transdermal delivery performance using excised mouse skin and compared with a 2% AS suspension. The suspension was prepared by dispersing bulk AS powder in a DMSO–glycerol–water mixture (5:28.5:66.5, v/v), followed by 5 min ultrasonication at 40 kHz.

Male Kunming mice (25 ± 10 g) were anesthetized in a KT-106 sealed induction chamber (Anhui, China) using a mixture of 4% isoflurane and oxygen (flow rate: 0.5 L/min) for 3 min, until the loss of the righting reflex. Subsequently, euthanasia was performed via cervical dislocation, and death was confirmed by pupil dilation, absence of spontaneous respiration, and cessation of cardiac activity. Dorsal skin was excised and depilated, and subcutaneous adipose tissue was removed. The full-thickness skin was mounted on Franz diffusion cells (TK-24BL, Beijing, China) with the stratum corneum facing the donor compartment.

Each donor compartment received 1 mL test formulation (hydrogel or suspension), while the receptor compartment contained 8 mL ethanol–saline mixture (20:80, v/v) maintained at 37°C ± 2°C. Receptor medium aliquots (5 mL) were sampled at 0.5, 1, 2, 4, 6, 8, 10, and 12 h, with immediate replacement of equal pre-warmed fresh medium. Post-experiment, the residual formulation was removed from skin surfaces. The skin was minced, sonicated in methanol (15 min, 40 kHz), and centrifuged (10,000 × g, 10 min) to isolate drugs retained in the skin (Qs, μg·cm^-2^). Drug quantification was performed using a UHPLC system with a C18 column (acetonitrile: water = 24:76 v/v, 1 mL/min, λ = 205 nm).

Cumulative permeated drug per unit area (Q_n_, μg·cm^-2^) was plotted against time. Steady-state flux (J_s_, μg·cm^-2^·h^-1^) was calculated as the slope of the linear Q_n-t_ regression. Lag time (T_lag_, h) was determined by extrapolating the linear regression to the time axis. Key parameters were calculated as follows:

$$Q_{n}=\frac{C_{n}V+\sum_{i=1}^{n-1}C_{i}V_{i}}{A},$$

where A denotes the effective diffusion area (cm^2^), V is the receptor volume (mL), V_i_ represents the volume after each sampling time, and C_i_ represents the drug concentration at *i*th sampling (μg/mL). Data are expressed as the mean ± SD (n = 3). The results were analyzed using a one-way analysis of variance (ANOVA) with SPSS (IBM^®^ SPSS^®^ Version 27), and *p < 0.05 was considered statistically significance.

### 2.8 Stability test

The freshly prepared 2% AS hydrogel was sealed in vials and stored at ambient temperature for 3 months. The sample appearance and morphology were checked per month. At the end of 3 months, the viscosity was measured using a rheometer.

## 3 Results

### 3.1 Gelation behavior and critical gelation concentration

**FIGURE 2:**
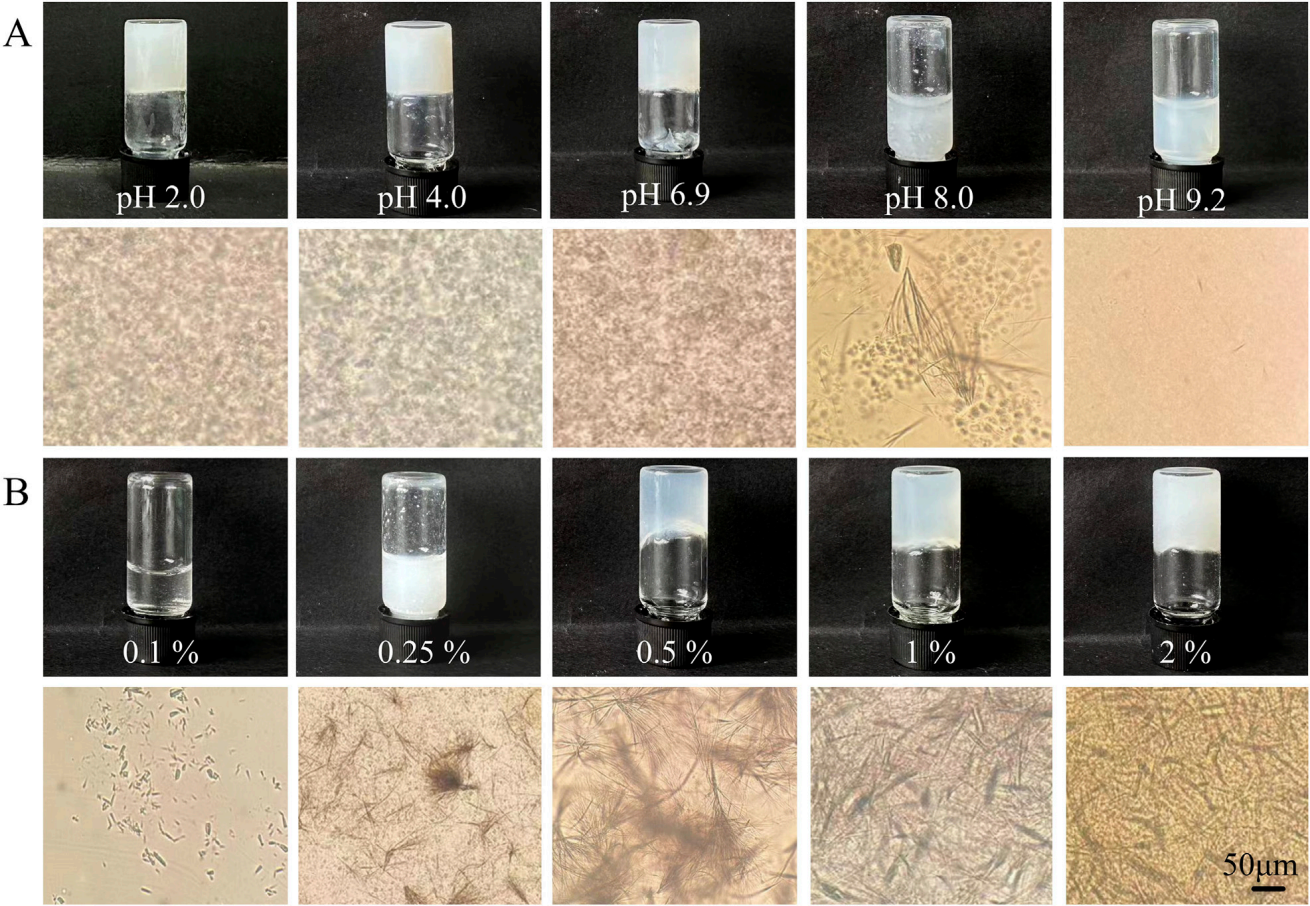
Appearance and microscopic images of AS in **(A)** various solvent systems or **(B)** glycerol–water mixtures at different drug levels.

The anti-solvent dispersion approach was employed to assess the gelation of AS in various solvents, and the outcomes for different AS gels are illustrated in Figure 2. At a fixed AS concentration of 2%, gel formation was evident in aqueous solutions at pH 2.0, 4.0, or 6.9 and a glycerol–water (3:7, v/v) mixture. All formed gels appeared pale white with numerous long, entangled fibers under microscopy. Drug precipitates were observed in aqueous solutions at pH 8.0, but these turned translucent in PBS at pH 9.2 (Figure 2A).

Considering biostability and biocompatibility requirements for biomedical and cosmetic applications, the glycerol–water mixed solvent was selected for further investigation. In this solvent system (Figure 2B), gelation was only observed when the AS concentration was ≥0.5%, thus establishing the critical gelation concentration (CGC) of the AS self-supporting hydrogel as 0.5% in this system. Below the CGC, suspensions with bundled short fibers were obtained. However, the connected long fibers in AS hydrogels decreased in length but increased in density with higher drug levels, indicating improved dispersion and connection in the 3D network of the gels.

### 3.2 Morphological characterization

**FIGURE 3:**
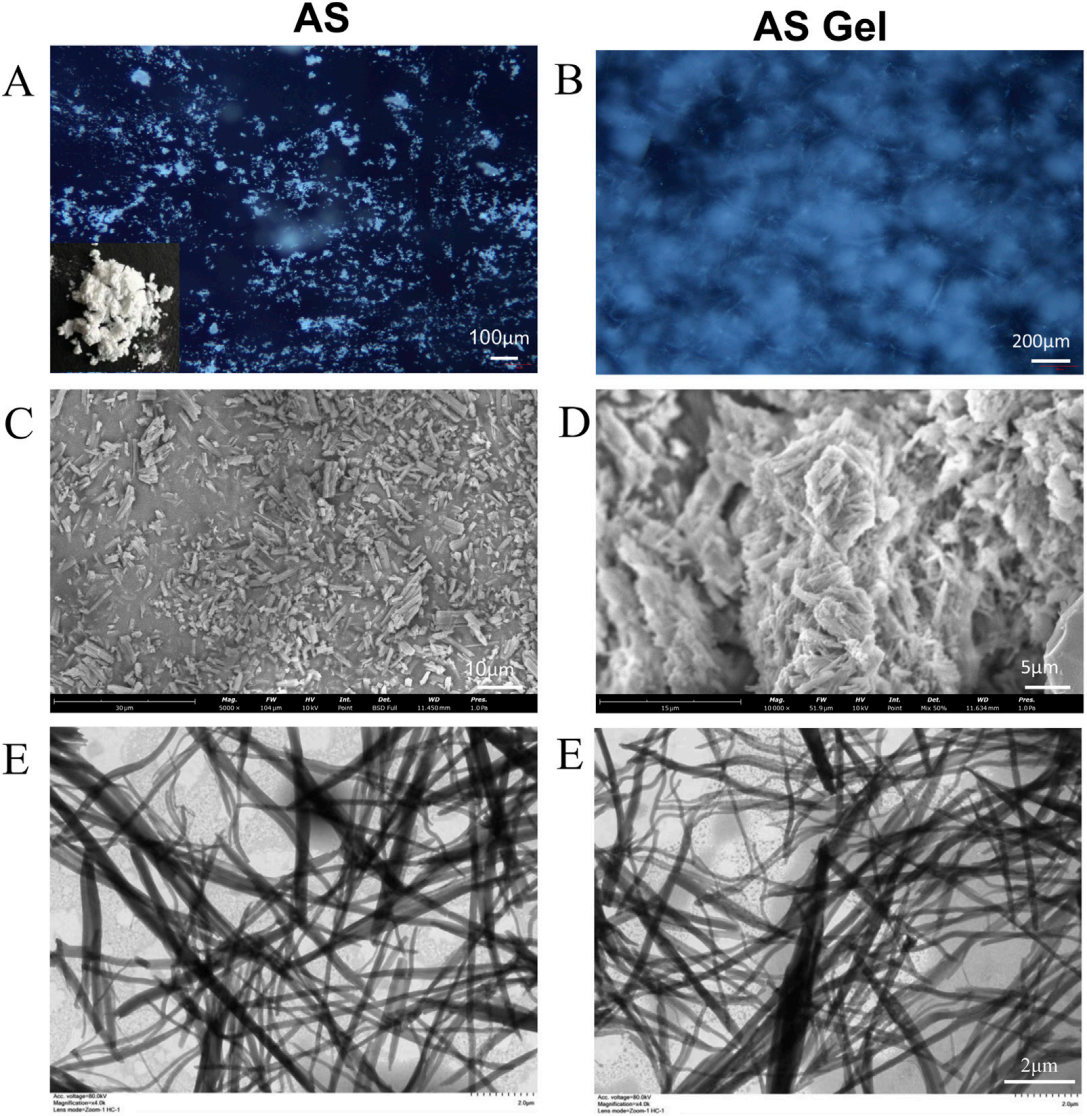
Images of powder and dry gel/hydrogel of AS under different microscopes. **(A, B)** PLM. **(C, D)** SEM. **(E)** TEM.

The morphology and structural characteristics of the obtained AS hydrogel were examined. The bulk AS is a white powder with a short-rod shape and birefringence, as shown in Figures 3A, C. In contrast, the freshly prepared AS hydrogel (Figure 3B) exhibits no obvious birefringence effect. The corresponding xerogel consists of large blocks composed of highly aggregated and partly fused pieces and bars, with lengths not exceeding 5 μm (Figure 3D). TEM observations revealed the presence of numerous interwoven nanofibers within the AS hydrogel, with lengths extending up to 10 μm and widths of approximately 100 nm (Figure 3E).

### 3.3 Physicochemical characterization

**FIGURE 4:**
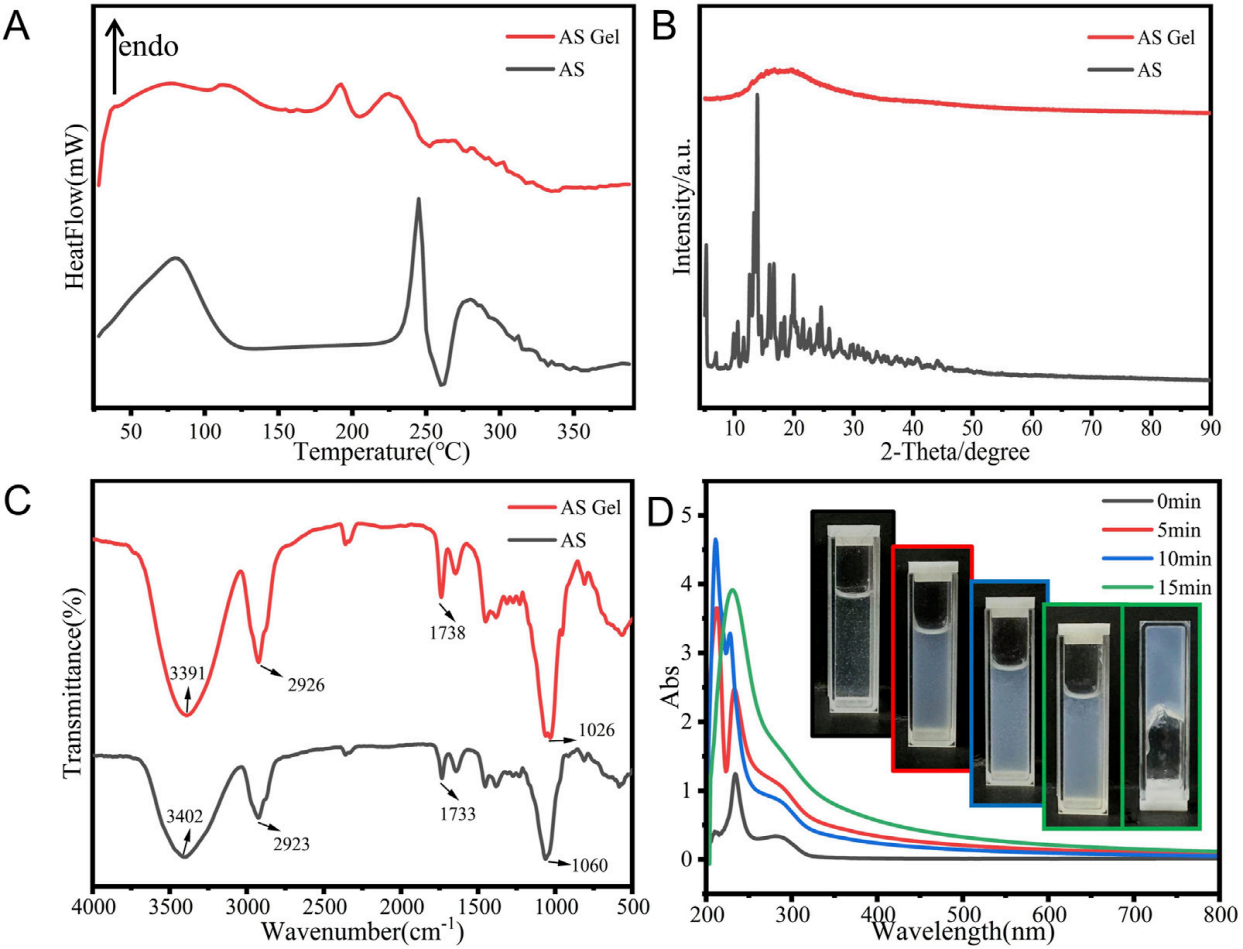
**(A)** DSC, **(B)** PXRD, **(C)** FTIR, and **(D)** UV–VIS spectra of the bulk AS and AS gels.

Multiple analysis methods were used to further elucidate the self-assembly process and underlying mechanism of AS gelation. The crystalline nature of the AS xerogel was confirmed by characteristic melting peaks at 245°C (Figure 4A) (Wannasarit et al., 2019) and multiple diffractogram peaks at a 2θ range of 10°–25° (Figure 4B). The loss of drug crystallinity was indicated by a single halo in the X-ray diffractogram and a weaker melting peak at a lower temperature in the DSC curve (Figures 4A, B).

The FTIR patterns of the bulk drug and xerogel are presented in Figure 4C. For the AS gel, hypsochromic shifts were observed in the O–H stretching (from 3,402 cm^-1^ to 3,391 cm^-1^), C–O–C stretching (from 1,455 cm^-1^ to 1,450 cm^-1^), and C=C stretching vibration (from 1,060 cm^-1^ to 1,026 cm^-1^); a bathochromic shift was observed in the C–C stretching (from 1,733 cm^-1^ to 1,738 cm^-1^). These shifts suggest the formation of potential intermolecular hydrogen bonds between the polyhydroxyl and carbonyl groups of AS molecules.

The time-dependent UV–VIS spectral evolution of the 0.5% AS hydrogel (Figure 4D) reveals critical insights into its supramolecular assembly dynamics. The initial shifts in absorption peaks (209→211→212 nm and 233→234→228 nm) within the first 10 min suggest progressive π–π stacking and intermolecular charge transfer, consistent with the formation of ordered molecular aggregates. The subsequent shift of the 228 nm peak back to 233 nm at 15 min, accompanied by the disappearance of the 212 nm peak, indicates a structural reorganization into a stabilized hydrogel network. This biphasic behavior aligns with reported mechanisms of triterpenoid self-assembly, where metastable intermediates (e.g., J-aggregates) transition to thermodynamically favored H-aggregates under supersaturated conditions (Greciano et al., 2020). The observed hyperchromic effect (peak intensity increase) further corroborates enhanced chromophore alignment via hydrophobic clustering (Dehkordi et al., 2012). These spectral signatures collectively validate the hierarchical assembly pathway proposed in our MD simulations.

The initial blueshift (Colín et al., 2023) and the subsequent redshift (Lin et al., 2024) suggest a transition from a dispersed state to a more aggregated state with enhanced π–π stacking within the hydrogel network. Throughout this process, the peak intensity surged initially, with a slight dip upon gelation, indicating the progressive establishment and stabilization of a supersaturated state within the supramolecular gel system.

Visually, the system underwent a transformation: starting from a clear, fluid state with minimal suspended particles, it gradually became turbid and semi-transparent, acquiring a viscous texture, and ultimately solidified into a hydrogel that resists flow upon inversion. These observations confirm that the gelation of AS in the aqueous medium is a dynamic assembly of drug molecules.

### 3.4 Molecular dynamics simulations

**FIGURE 5:**
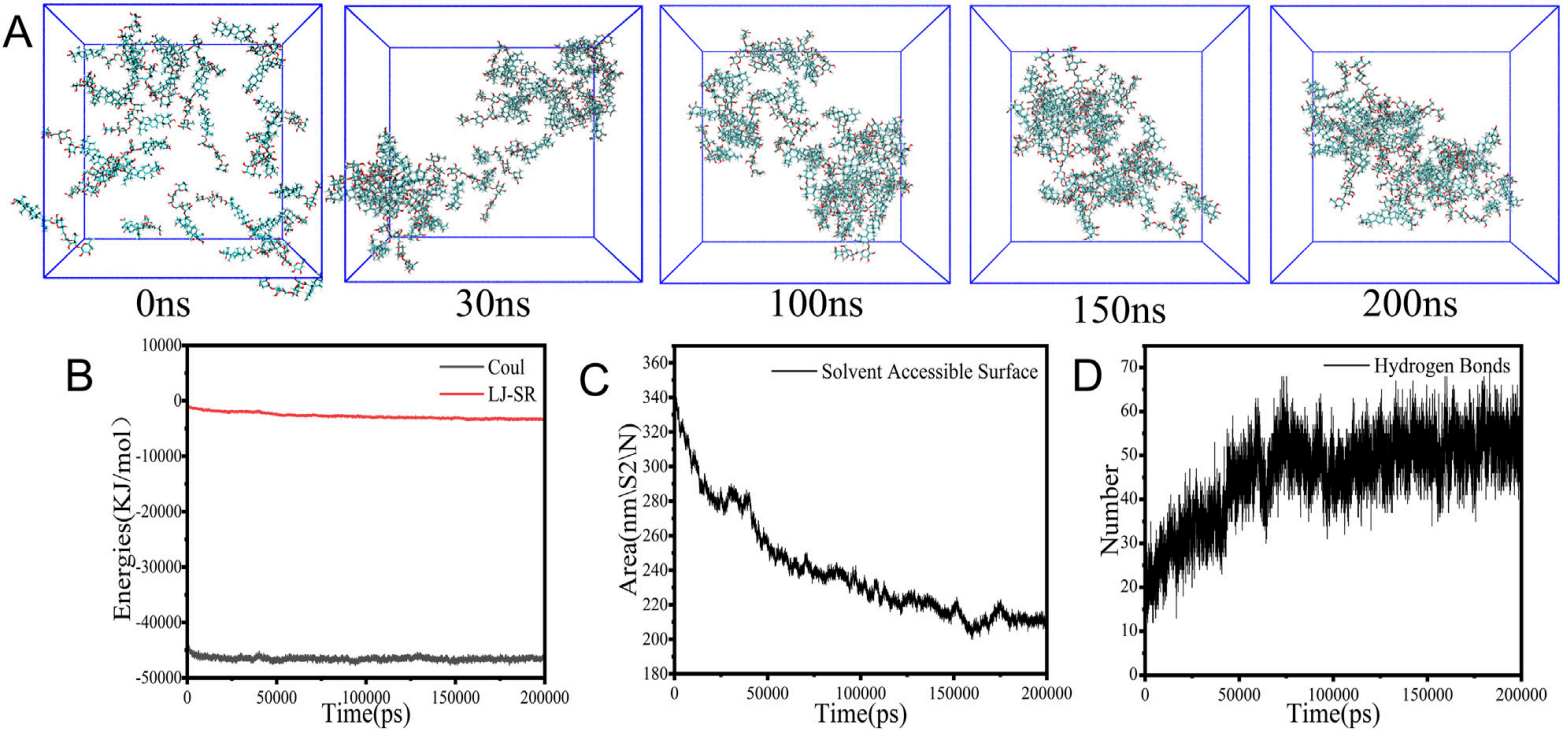
Aggregation and parameters in MD simulation. **(A)** Snapshots. **(B)** Energy values. **(C)** SASA values. **(D)** Hydrogen bond numbers.

MD simulations were performed to meticulously examine the self-assembly dynamics of AS molecules in aqueous medium, with the findings summarized in Figure 5. Initially dispersed within the cubic box, the AS molecules began to aggregate within the first 30 ns and formed a compact structure by the 200 ns mark, as shown in Figure 5A. The Coulombic interaction strength was approximately 50-fold greater than the Lennard–Jones (LJ) interaction strength, denoted as LJ-SR (Figure 5B), highlighting the overwhelming influence of electrostatic forces over van der Waals forces during the assembly phase.

Concurrently, the solvent accessible surface area (SASA) steadily decreased from 340 nm^2^ to 200 nm^2^ (Figure 5C), and the count of hydrogen bonds surged from 20 to 55 within the first 100 ns of the simulation (Figure 5D), which indicated the formation of new intermolecular hydrogen bonds that stabilized the compact structure. These observations suggest that the aggregation of AS molecules is predominantly governed by hydrophobic interactions and π–π stacking (Lei et al., 2025).

### 3.5 Rheological properties

**FIGURE 6:**
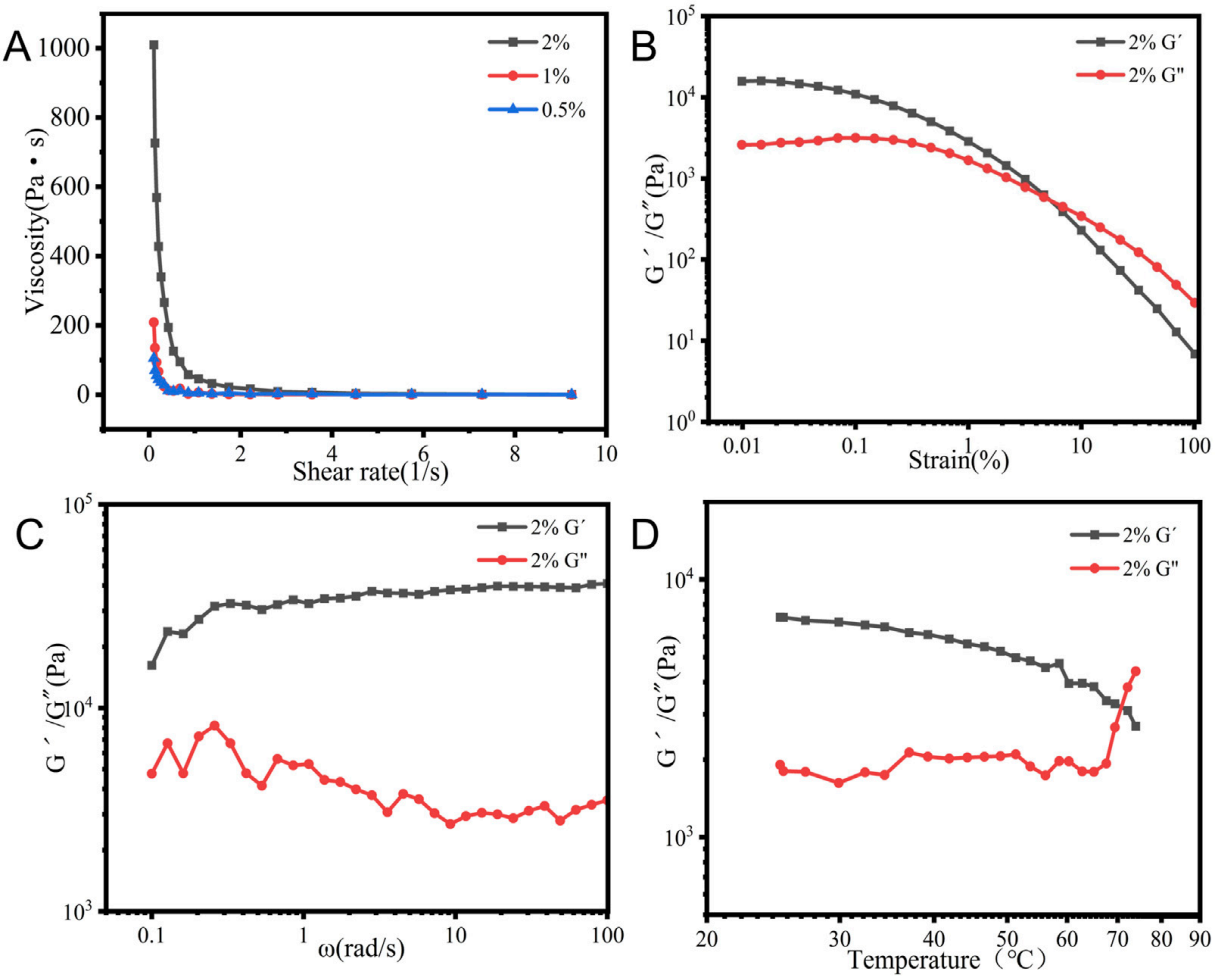
Rheological characteristics of 2% AS hydrogel in a glycerol–water system: **(A)** viscosity profiles. **(B)** Strain sweep. **(C)** Frequency sweep. **(D)** Modulus as a function of temperature.

The rheological properties of AS gel were investigated using a rheometer test, and the research results are shown in Figure 6. As depicted in Figure 6A, the viscosity of the AS hydrogel increased proportionally with higher drug concentrations in the glycerol–water medium. All hydrogel samples showed a rapid decrease in viscosity with increasing shearing rate, suggesting shear-thinning characteristic and non-Newton fluid nature. Both G′ and G″ gradually decreased as the shear strain percentage increased (Figure 6B), with a crossover at approximately 4.6%, indicating the occurrence of a gel-to-sol transition. This critical value of γ suggested the yield strength of AS hydrogel as a viscous–plastic medium. The tested 2% AS hydrogel exhibited a solid-like behavior (Figure 6C), with the storage modulus (G′) dominant over the loss modulus (G″) throughout the study range of ω, suggesting its elastic property. During the heating process (Figure 6D), G′ gradually decreased, while G″ exhibited fluctuations, followed by a sharp increase and a crossover with G′ at 70°C. This result indicates a complete disintegration of the 3D network structure of AS hydrogel at this temperature.

### 3.6 Skin-permeation performance

**FIGURE 7:**
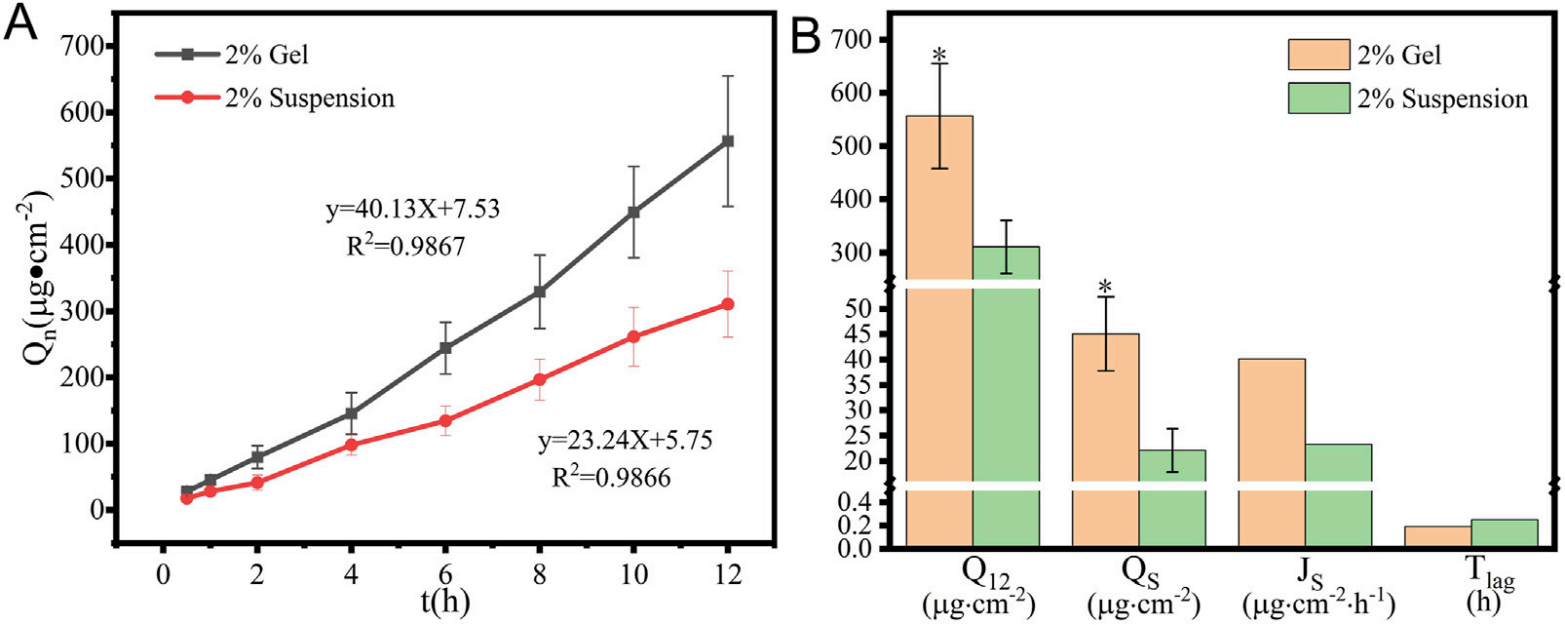
*In vitro* permeation **(A)** profiles and **(B)** key parameters of 2% AS suspension and hydrogel (x ± SD, n = 3,*p < 0.05, versus the suspension group).

The cumulative drug-permeation profiles on mice skin are depicted in Figure 7A. Drug penetration through mouse skin was significantly higher for the hydrogel than for the suspension at the same AS level and within the same solvent system. The flux rate (J_s_) and cumulative permeated drug within 12 h (Q_12_) of the hydrogel formulation were 1.73- and 1.79-times higher than those of the suspension formulation. Additionally, the drug amount retained (Q_s_) in the tested skin of the hydrogel was 2.04-times higher than that of the suspension (Figure 7B). The results confirmed that the AS supramolecular hydrogel showed a superior permeation and retention performance on mice skin.

### 3.7 Storage stability

**FIGURE 8:**
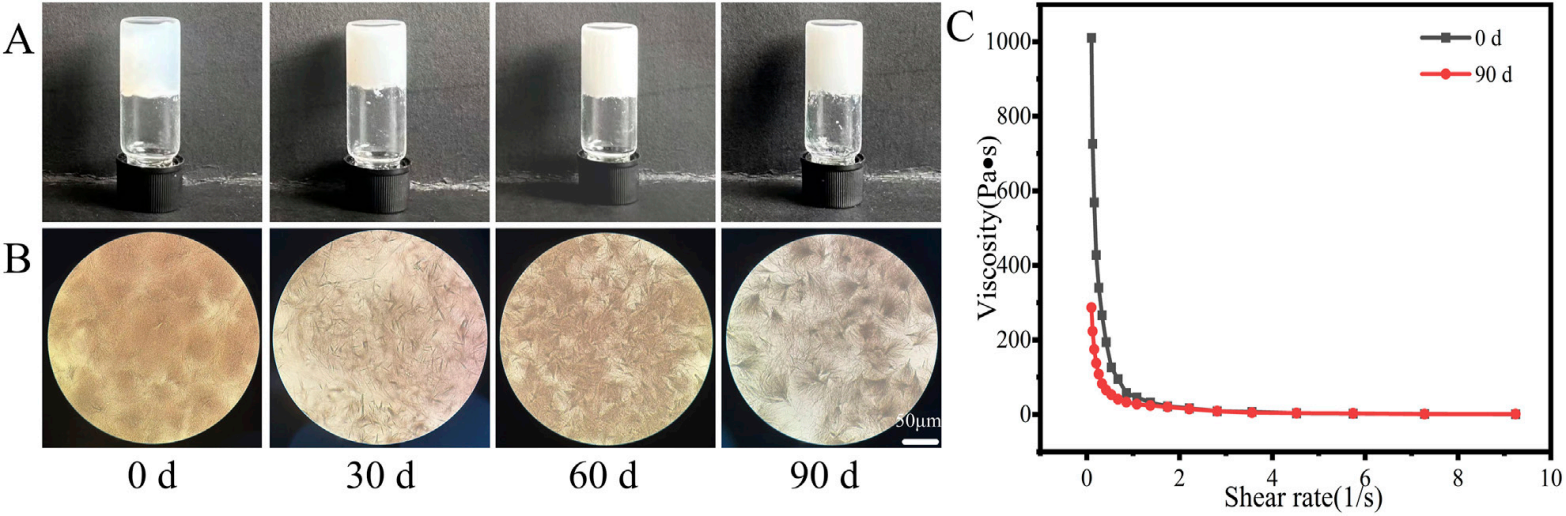
**(A)** Appearance, **(B)** micromorphology, and **(C)** viscosity of AS hydrogel after storage for 3 months.

As shown in Figure 8A, the AS hydrogel maintained its self-supporting state during storage under ambient conditions, with no obvious changes in appearance but with more large fiber bundles under microscopy (Figure 8B). At the end of the 90-day stability test, the viscosity of the hydrogel was two-thirds lower than that of the newly prepared hydrogel (Figure 8C), which was possibly due to fiber growth over time.

## 4 Discussions

This work establishes AS as a natural supramolecular gelator, expanding the repertoire of triterpenoid-based biomaterials. The pH-responsive gelation (CGC 0.5% w/v) aligns with reported glycoside self-assembly thresholds (Liu et al., 2023), while glycerol–water systems mitigate crystallization risks common in aqueous gels (Zhao et al., 2022). Notably, the hydrogel’s shear-thinning property enables skin application while retaining elasticity, which is critical for patient compliance.

Physiochemical characterization and MD simulations elucidate hierarchical assembly: initial electrostatic steering (Coul-SR dominance) nucleates clusters, followed by H-bond stabilization and π–π stacking consolidation—a mechanism paralleling saponin-based micelle formation (Mo et al., 2023).

Previous investigations have employed multiple strategies—including penetration enhancers, nanoemulsion-embedded hydrogels, and microneedles—to improve the transdermal permeability of AS (Wang et al., 2022). Although these systems demonstrated enhanced drug permeation (1.5- to 5-fold flux increase vs controls), critical limitations persist in suboptimal drug-loading capacities (<1% w/w), complex multi-step manufacturing, and production costs. These challenges highlight the need for simplified and cost-effective delivery platforms.

In the present study, the supramolecular hydrogel of AS molecules showed a superior permeation and retention performance on mouse skin, which is of special value for fully playing its role in managing dermatological conditions (Paolino et al., 2012). A similar enhanced skin permeation behavior was also observed in our previous report on breviscapine organogel (Namviriyachote et al., 2020), suggesting a potential benefit and application of the supramolecular gel system formed by the natural product gelators. The nanofibrous architecture enhances transdermal delivery through dual pathways: (1) supersaturation maintenance: amorphous AS in the hydrogel increases thermodynamic activity, consistent with nanoformulation-enhanced permeation (Chatterjee et al., 2022). (2) Stratum corneum modulation: glycerol plasticizes skin lipids (Fluhr et al., 2008), synergizing with nanofiber-induced hydration gradients to boost drug flux.

Supersaturated systems may cause spontaneous nucleation over time. This phenomenon is governed by the kinetic competition between nucleation and growth rates (Joshi and Sangamwar, 2018), where nucleation dominates at elevated supersaturation. The potential mitigation approaches to specific systems are expected to balance thermodynamic and kinetic factors. Solvent polarity and hydrogen-bonding capacity influence supersaturation thresholds (Kumar et al., 2023). Additives such as polymers or ions can also stabilize metastable phases or suppress undesirable polymorphs (Guo et al., 2016).

Compared to synthetic polymer gels, AS hydrogels offer inherent bioactivity (Bylka et al., 2014) and biodegradability, aligning with green chemistry principles. This work bridges phytochemistry and nanobiotechnology, providing a template for repurposing underutilized plant metabolites into functional biomaterials.

## 5 Conclusion

This study demonstrates the successful self-assembly of AS, a natural triterpene saponin, into a supramolecular hydrogel through an anti-solvent dispersion strategy. The hydrogel exhibits dual functional advantages: pH-responsive self-supporting gelation at a low critical concentration and enhanced skin-permeation efficiency. Mechanistic studies combining experimental characterization and molecular dynamics simulations revealed that hydrogen bonding and π–π stacking interactions drive the formation of an interwoven nanofiber network, which governs the hydrogel’s viscoelastic properties and drug release behavior.

The nanostructured hydrogel addresses AS’s limitations in solubility and bioavailability while retaining its intrinsic bioactivity. This work provides a mechanistically robust approach to repurpose plant-derived saponins into functional biomaterials, bridging traditional phytochemistry with contemporary nanobiotechnology for sustainable drug delivery solutions. Future work will focus on expanding therapeutic applicability by encapsulating combination drugs and validating efficacy in preclinical models of dermatological disorders.

## Data Availability

The original contributions presented in the study are included in the article/supplementary material; further inquiries can be directed to the corresponding authors.
