# Supplementary figures and images for: Supramolecular nanofibers of natural asiaticoside for self-supporting gelation and enhanced transdermal delivery
Source: Front Bioeng Biotechnol. 2025 May 2;13:1589865. doi: 10.3389/fbioe.2025.1589865 (PMC12081450; doi:10.3389/fbioe.2025.1589865)

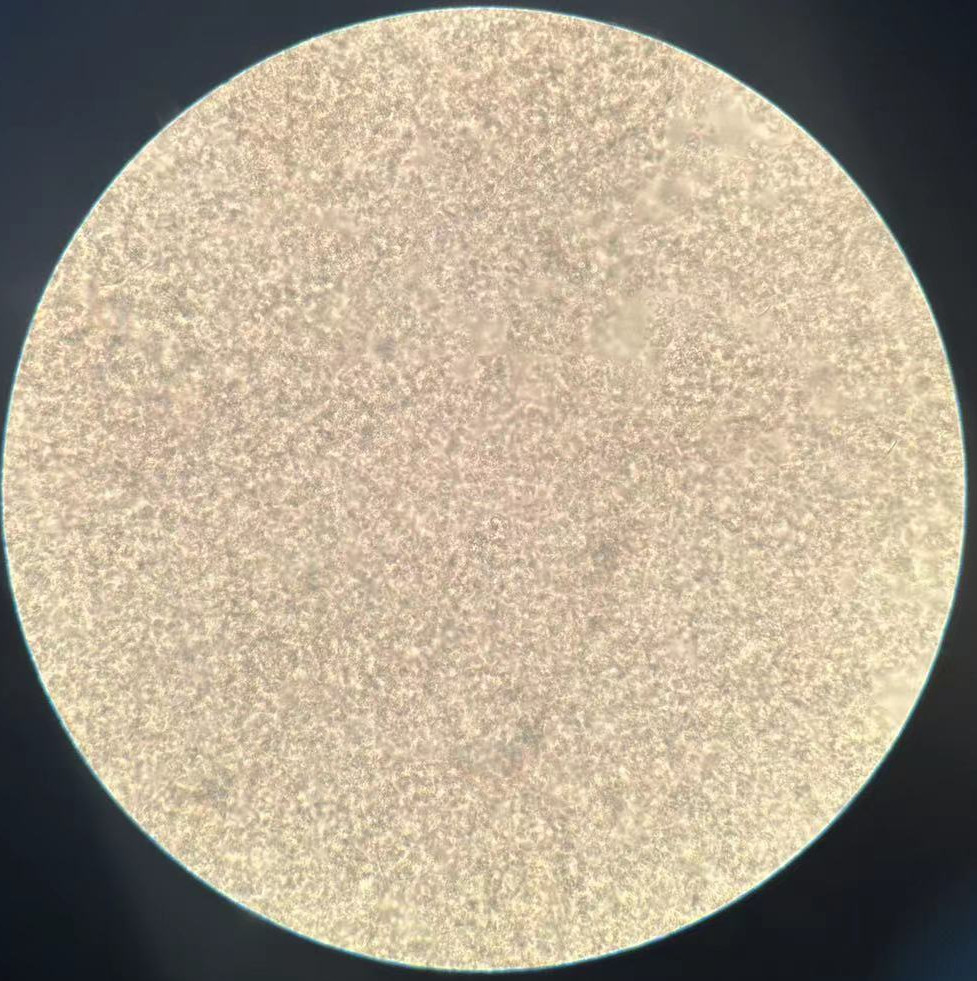

Supplement: Supplementary file 1 [file DataSheet1.zip › original image/Figure 1A -pH 2.0 (microscopy).jpg]

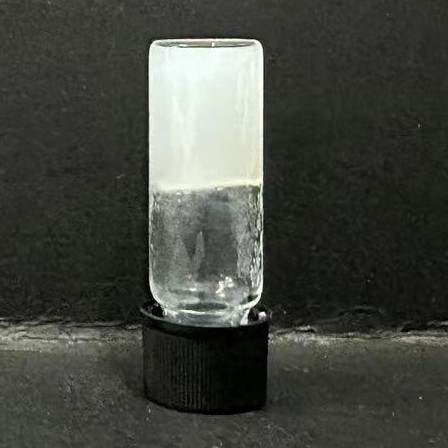

Supplement: Supplementary file 1 [file DataSheet1.zip › original image/Figure 1A -pH 2.0.jpg]

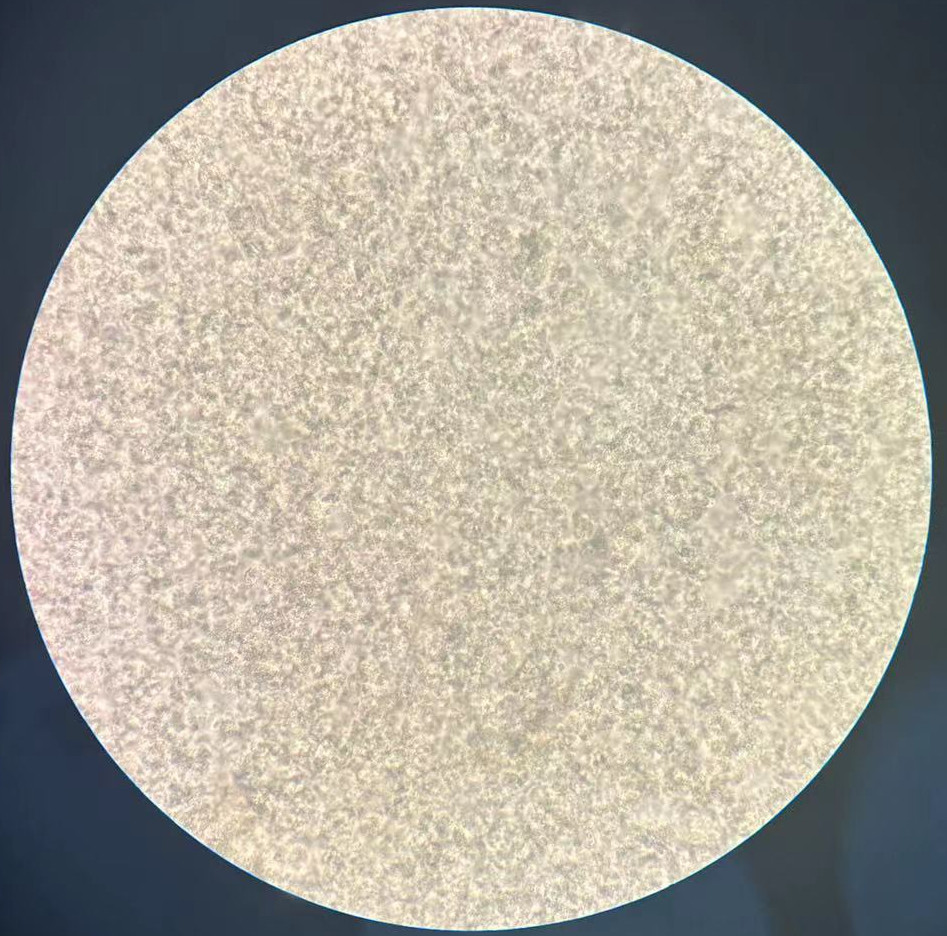

Supplement: Supplementary file 1 [file DataSheet1.zip › original image/Figure 1A -pH 4.0 (microscopy).jpg]

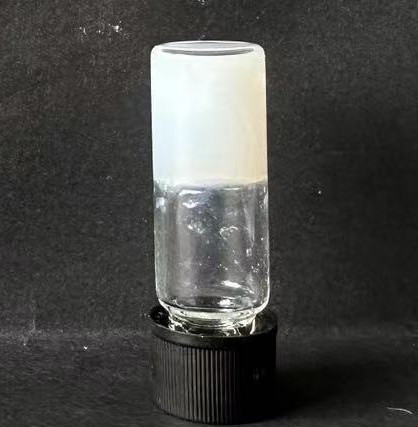

Supplement: Supplementary file 1 [file DataSheet1.zip › original image/Figure 1A -pH 4.0.jpg]

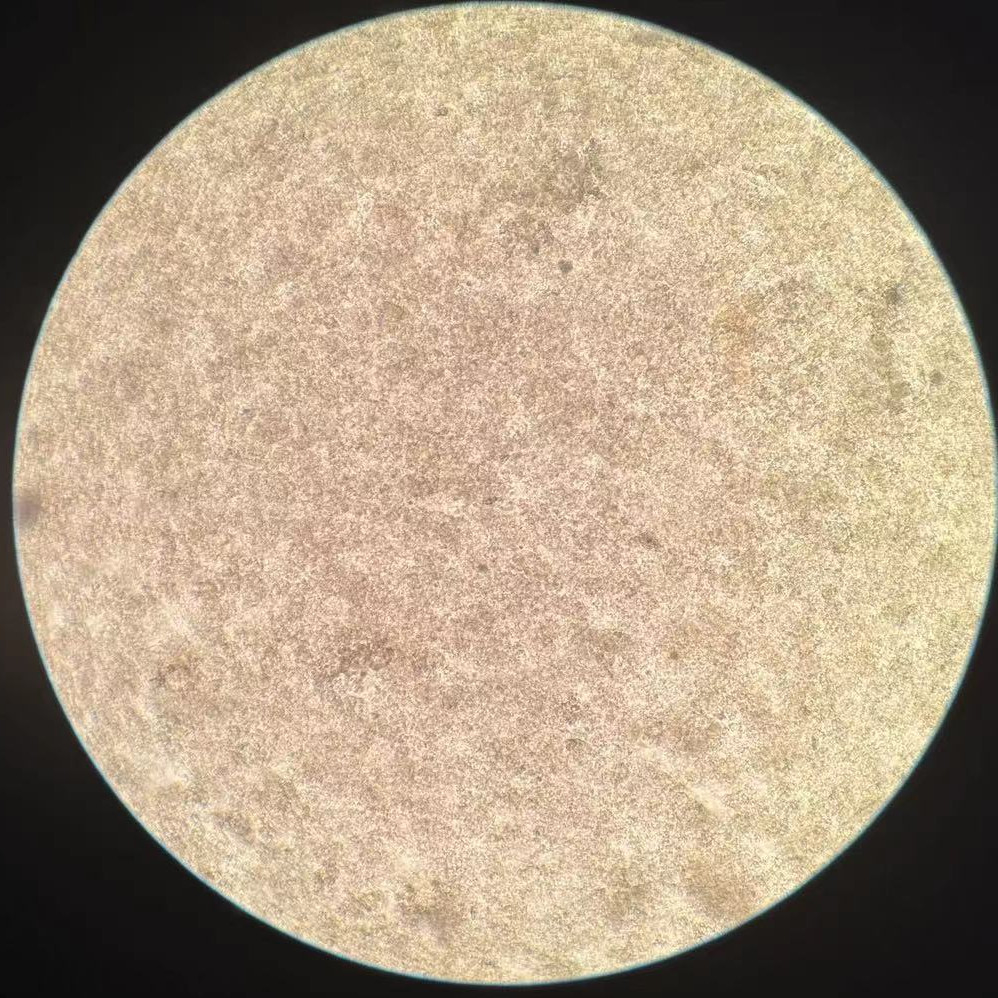

Supplement: Supplementary file 1 [file DataSheet1.zip › original image/Figure 1A -pH 6.9 (microscopy).jpg]

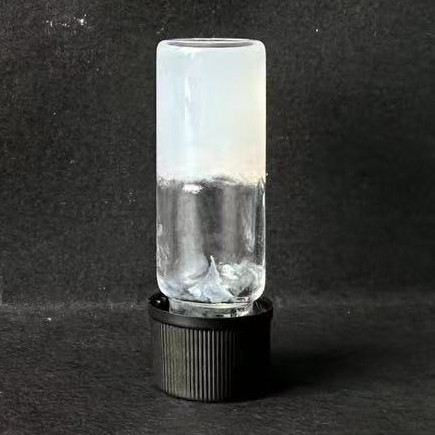

Supplement: Supplementary file 1 [file DataSheet1.zip › original image/Figure 1A -pH 6.9.jpg]

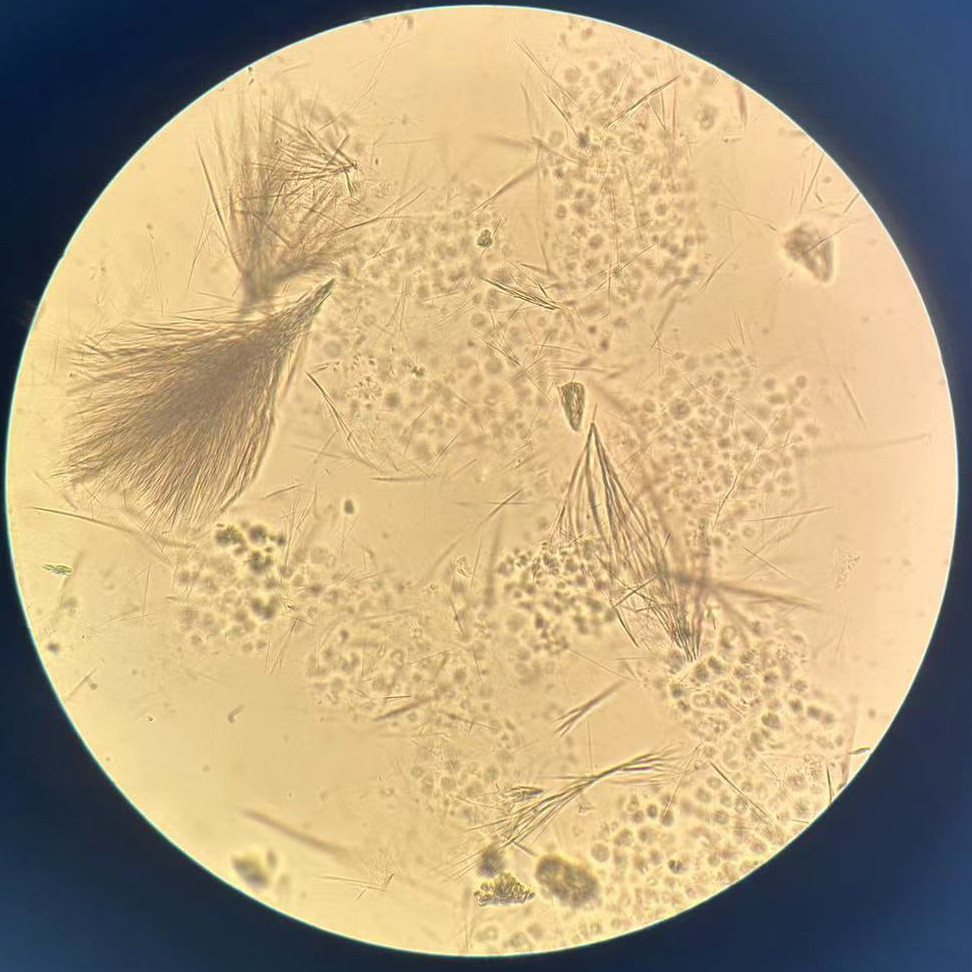

Supplement: Supplementary file 1 [file DataSheet1.zip › original image/Figure 1A -pH 8.0 (microscopy).jpg]

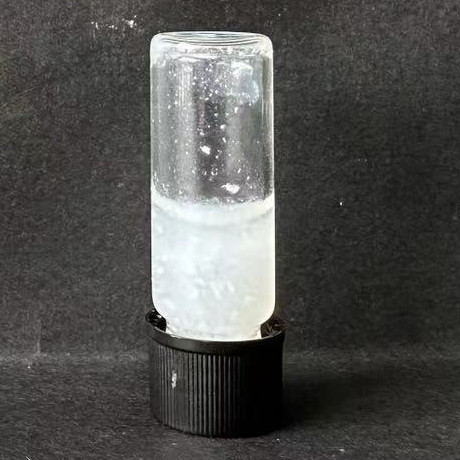

Supplement: Supplementary file 1 [file DataSheet1.zip › original image/Figure 1A -pH 8.0.jpg]

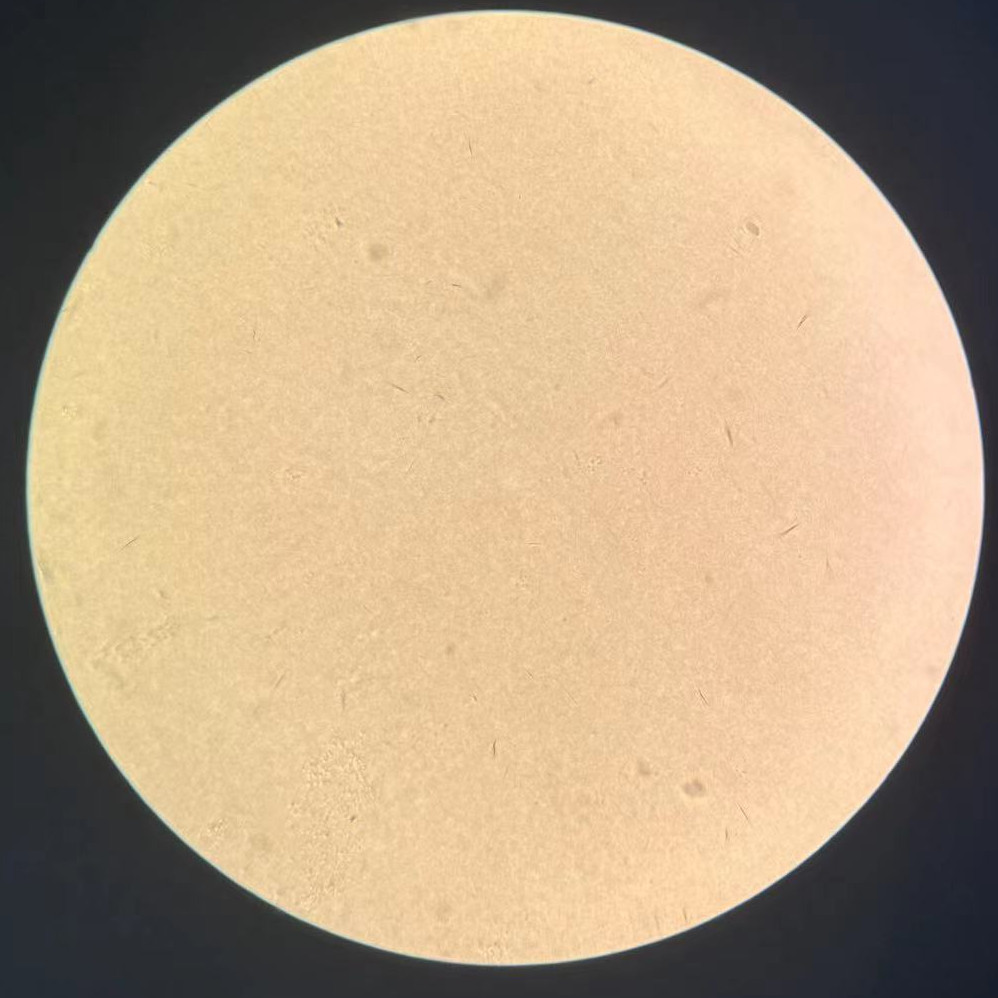

Supplement: Supplementary file 1 [file DataSheet1.zip › original image/Figure 1A -pH 9.2 (microscopy).jpg]

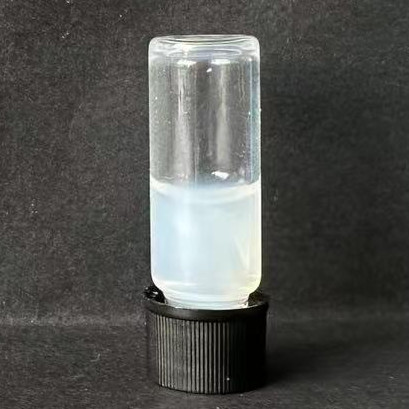

Supplement: Supplementary file 1 [file DataSheet1.zip › original image/Figure 1A -pH 9.2.jpg]

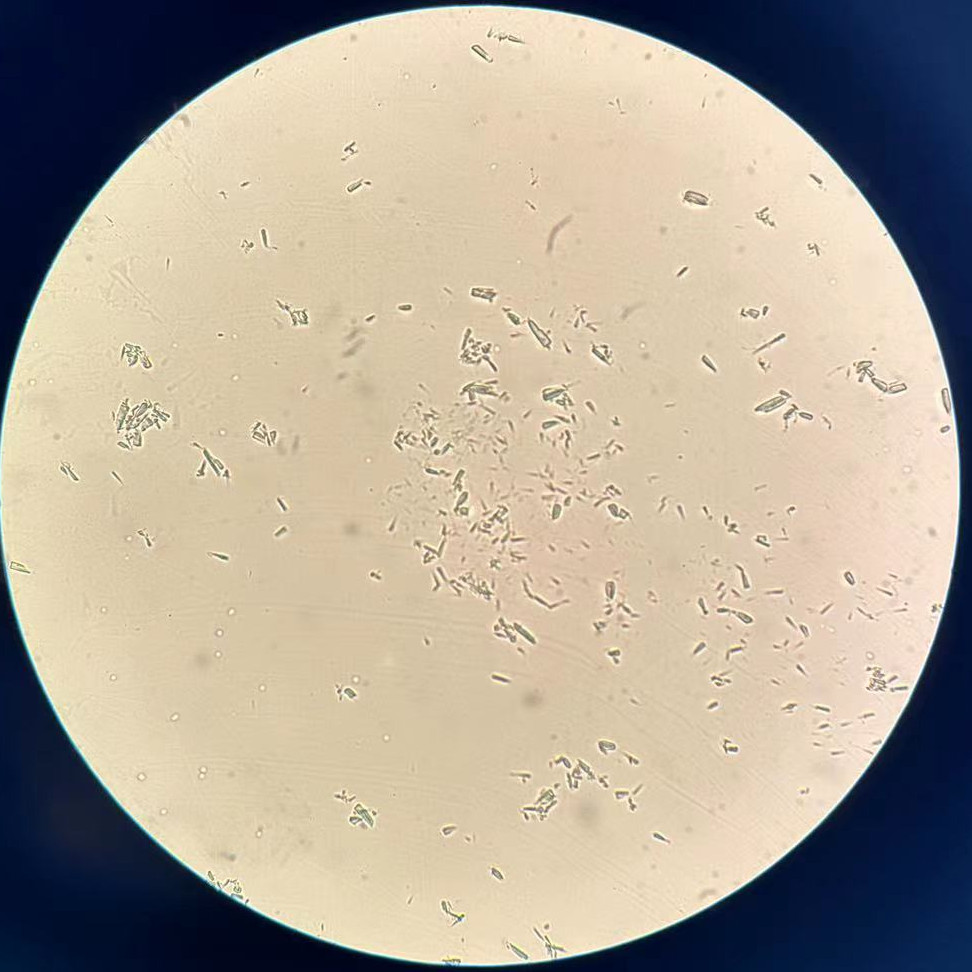

Supplement: Supplementary file 1 [file DataSheet1.zip › original image/Figure 1B-0.1%(microscopy).jpg]

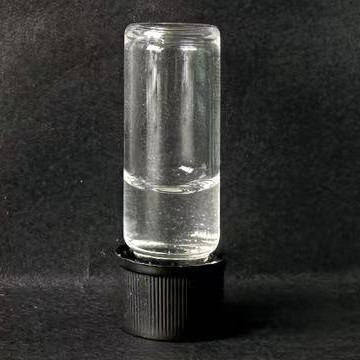

Supplement: Supplementary file 1 [file DataSheet1.zip › original image/Figure 1B-0.1%.jpg]

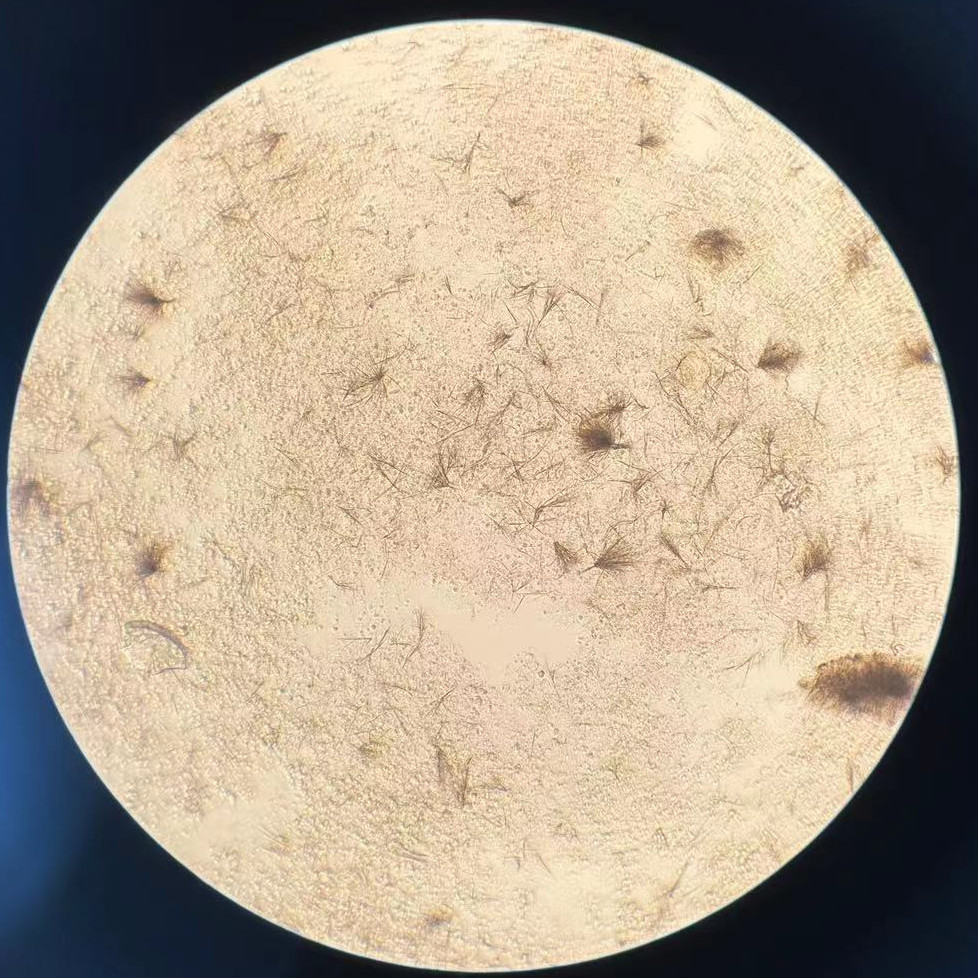

Supplement: Supplementary file 1 [file DataSheet1.zip › original image/Figure 1B-0.25%(microscopy).jpg]

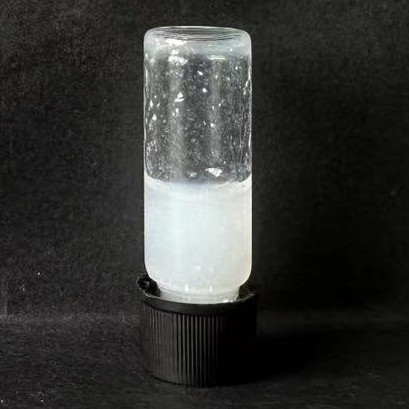

Supplement: Supplementary file 1 [file DataSheet1.zip › original image/Figure 1B-0.25%.jpg]

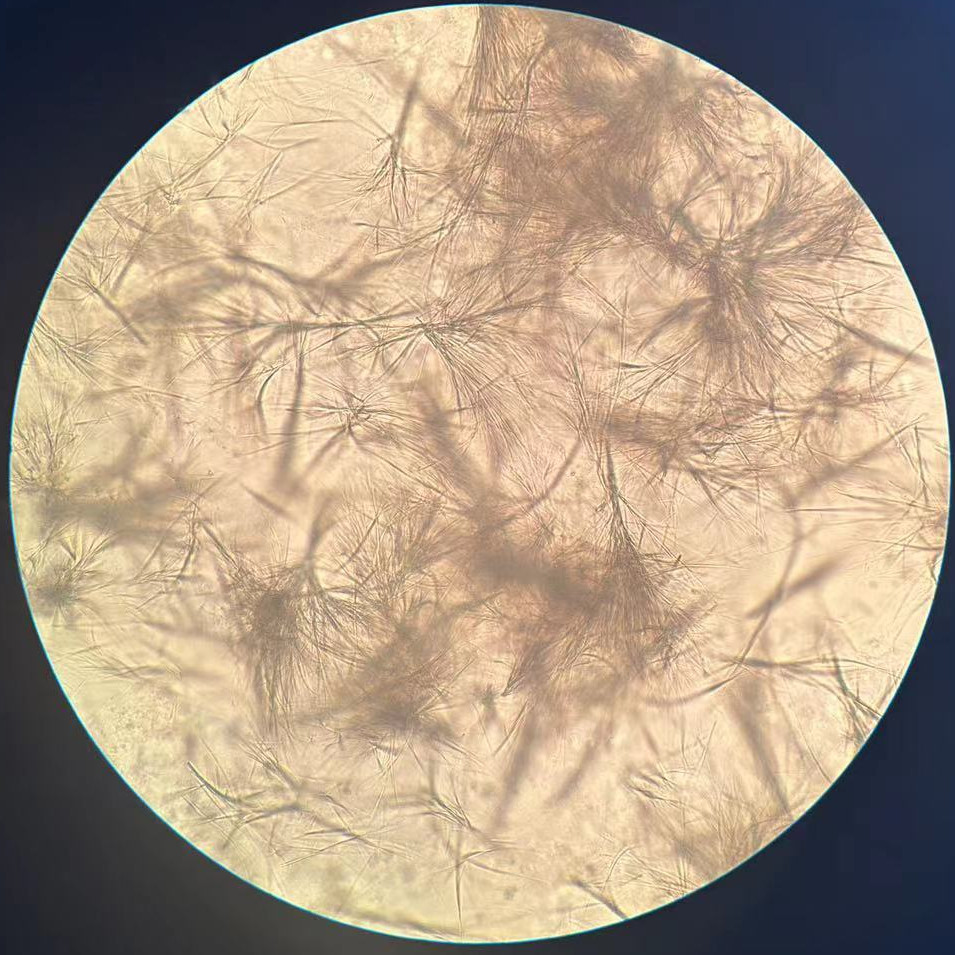

Supplement: Supplementary file 1 [file DataSheet1.zip › original image/Figure 1B-0.5%(microscopy).jpg]

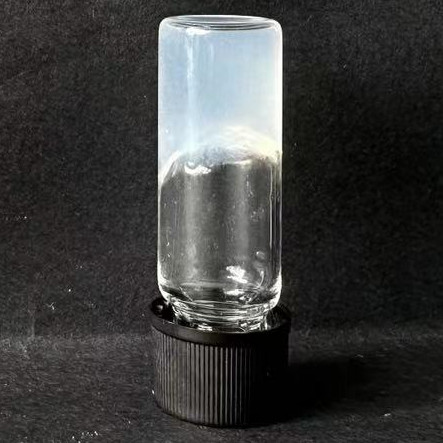

Supplement: Supplementary file 1 [file DataSheet1.zip › original image/Figure 1B-0.5%.jpg]

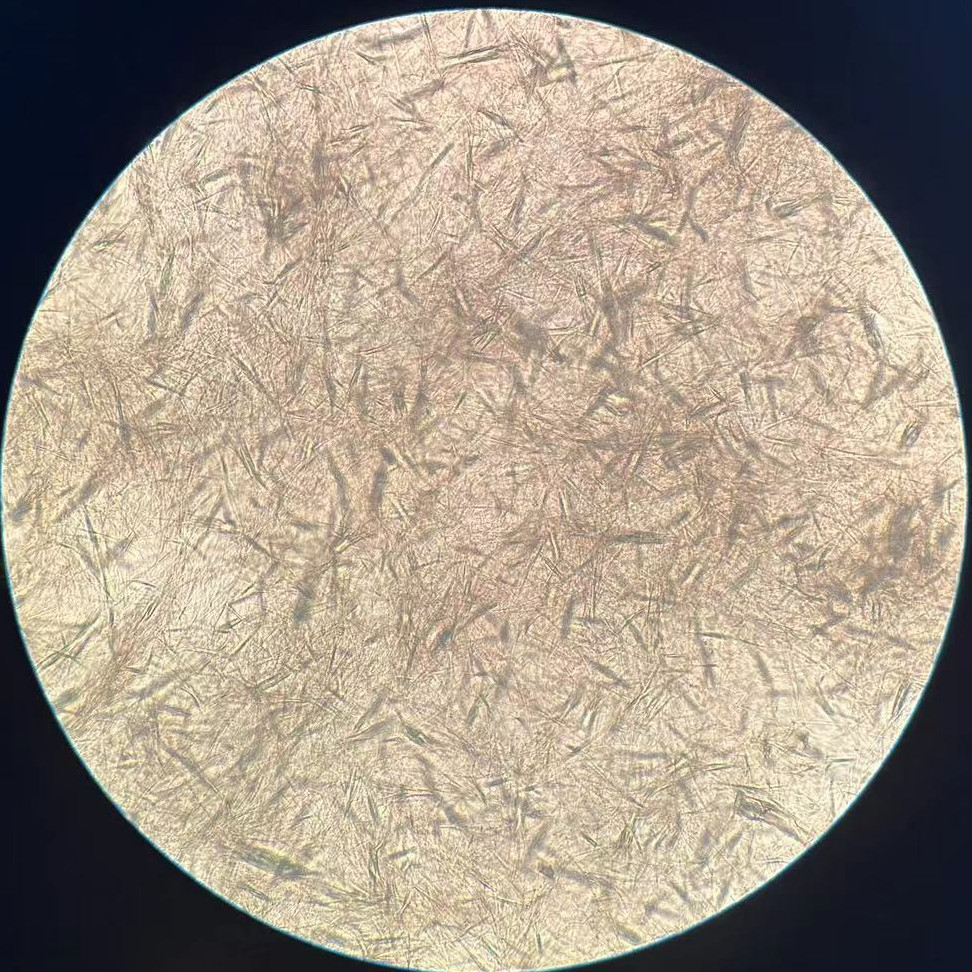

Supplement: Supplementary file 1 [file DataSheet1.zip › original image/Figure 1B-1%(microscopy).jpg]

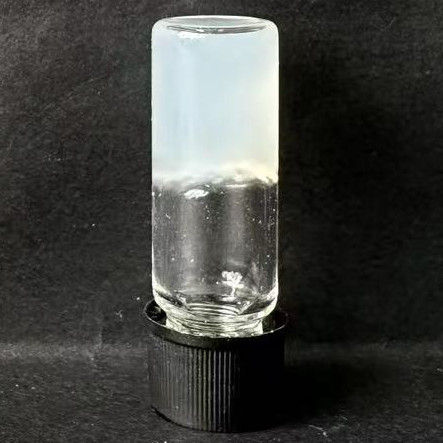

Supplement: Supplementary file 1 [file DataSheet1.zip › original image/Figure 1B-1%.jpg]

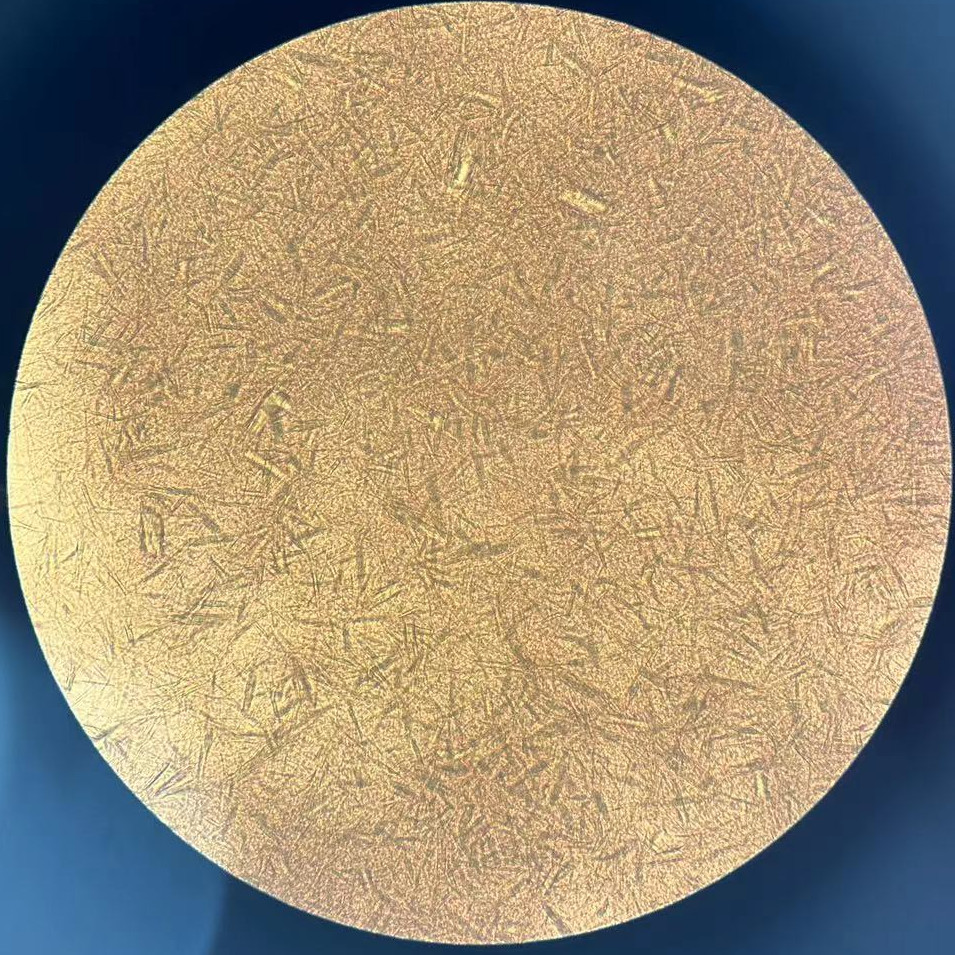

Supplement: Supplementary file 1 [file DataSheet1.zip › original image/Figure 1B-2%(microscopy).jpg]

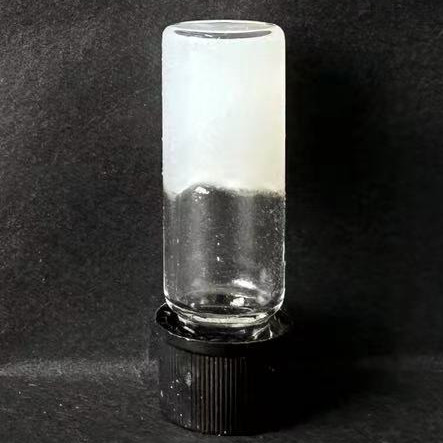

Supplement: Supplementary file 1 [file DataSheet1.zip › original image/Figure 1B-2%.jpg]

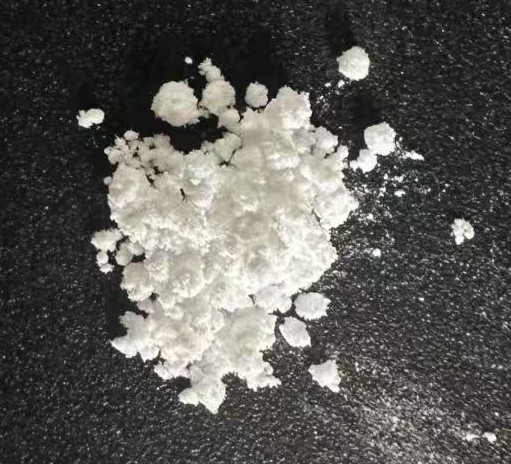

Supplement: Supplementary file 1 [file DataSheet1.zip › original image/Figure 2A-1.jpg]

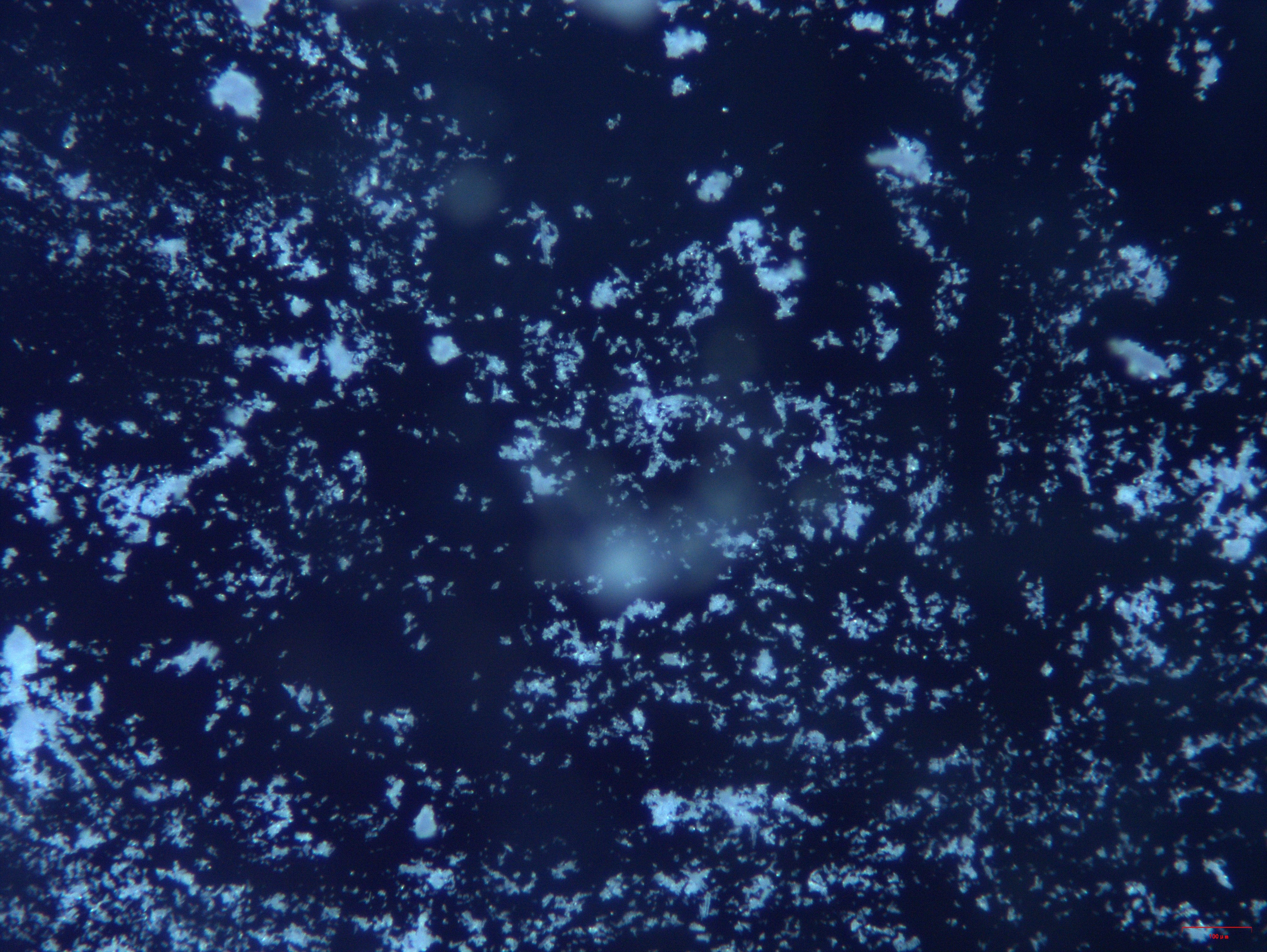

Supplement: Supplementary file 1 [file DataSheet1.zip › original image/Figure 2A.jpg]

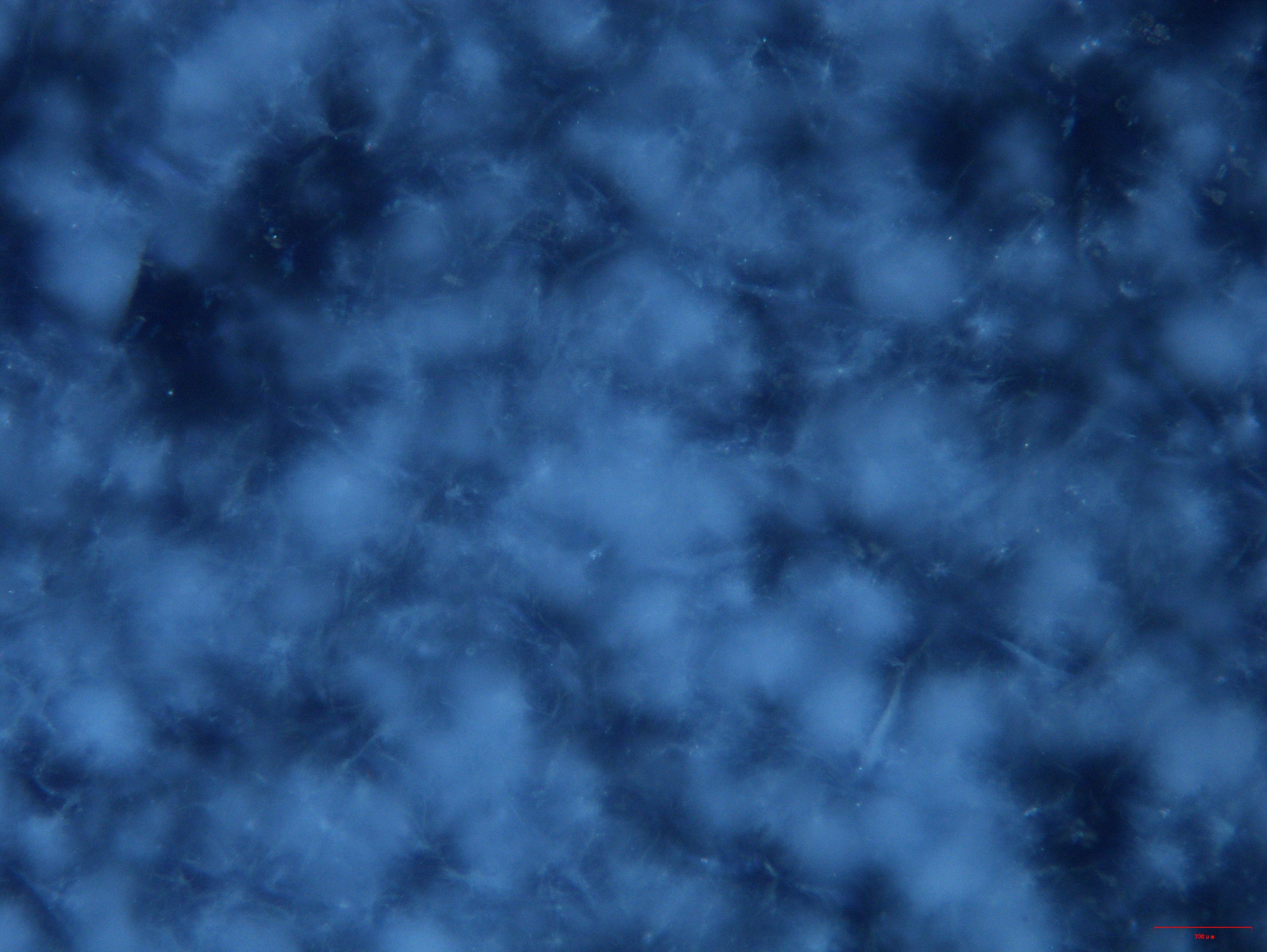

Supplement: Supplementary file 1 [file DataSheet1.zip › original image/Figure 2B.jpg]

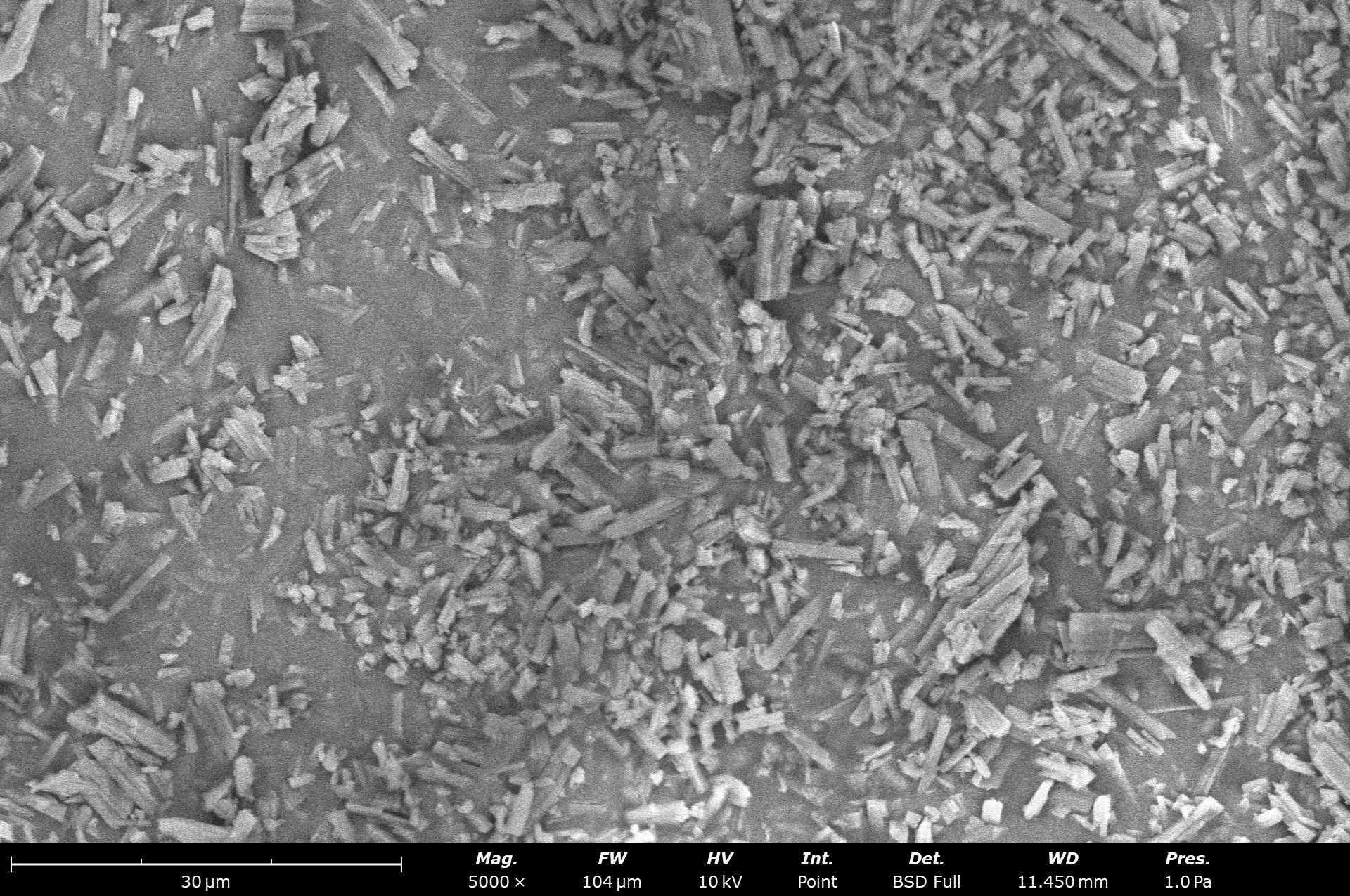

Supplement: Supplementary file 1 [file DataSheet1.zip › original image/Figure 2C.jpg]

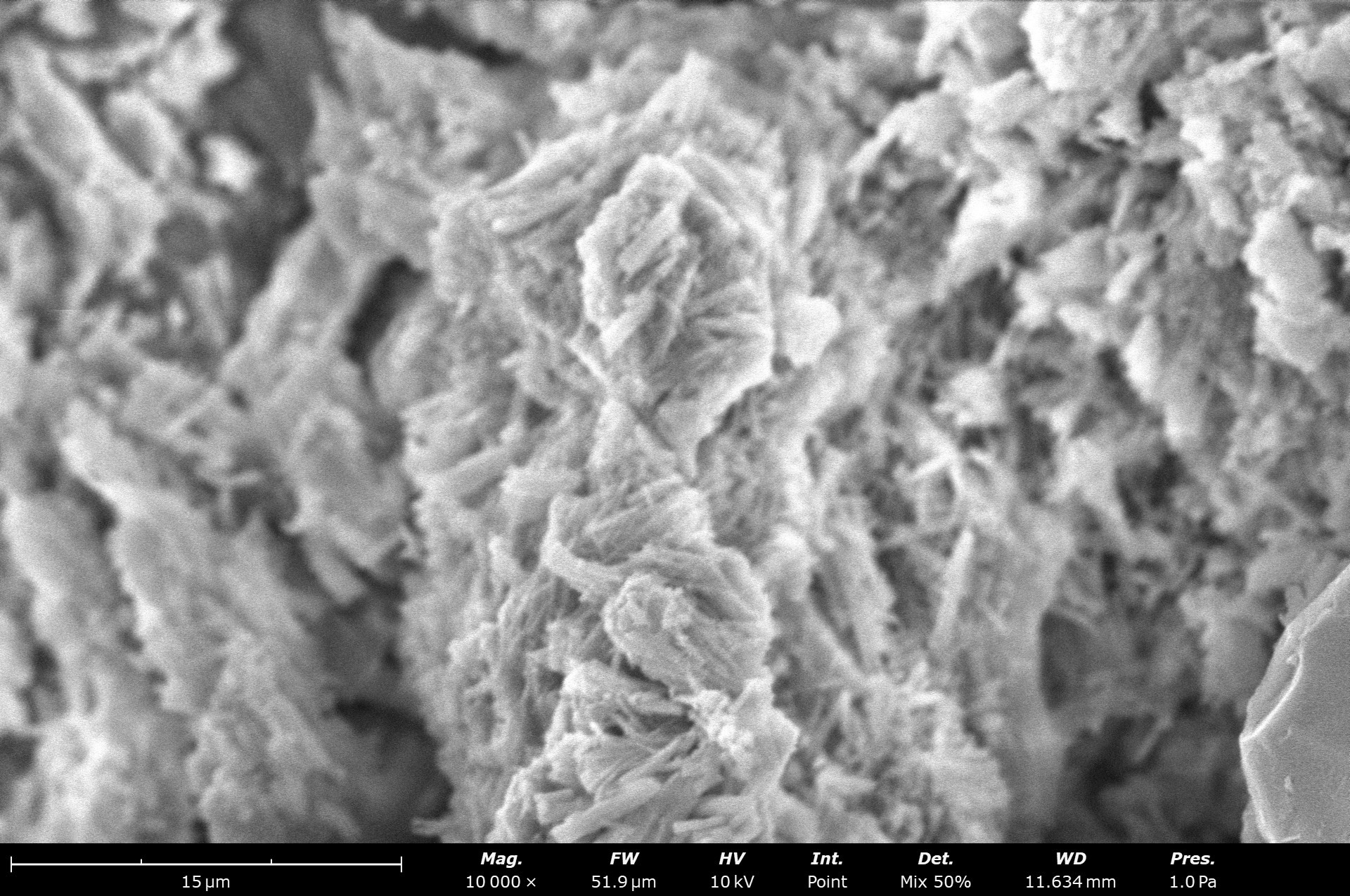

Supplement: Supplementary file 1 [file DataSheet1.zip › original image/Figure 2D.jpg]

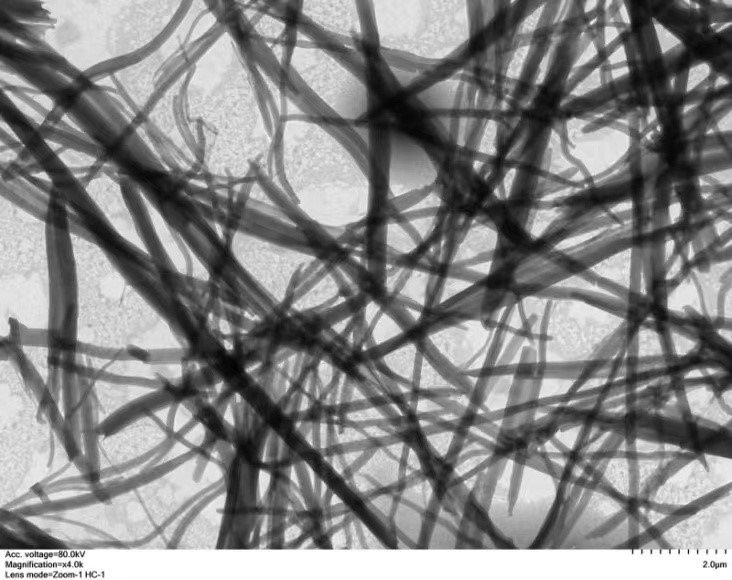

Supplement: Supplementary file 1 [file DataSheet1.zip › original image/Figure 2E-1.jpg]

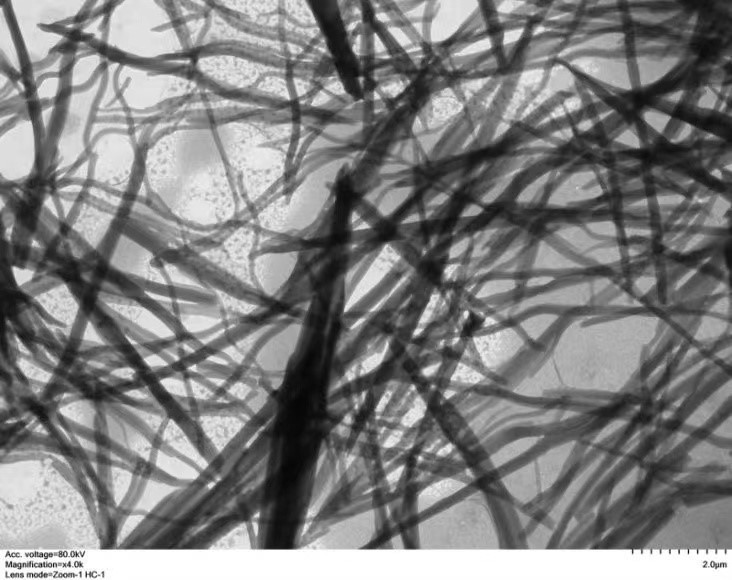

Supplement: Supplementary file 1 [file DataSheet1.zip › original image/Figure 2E-2.jpg]

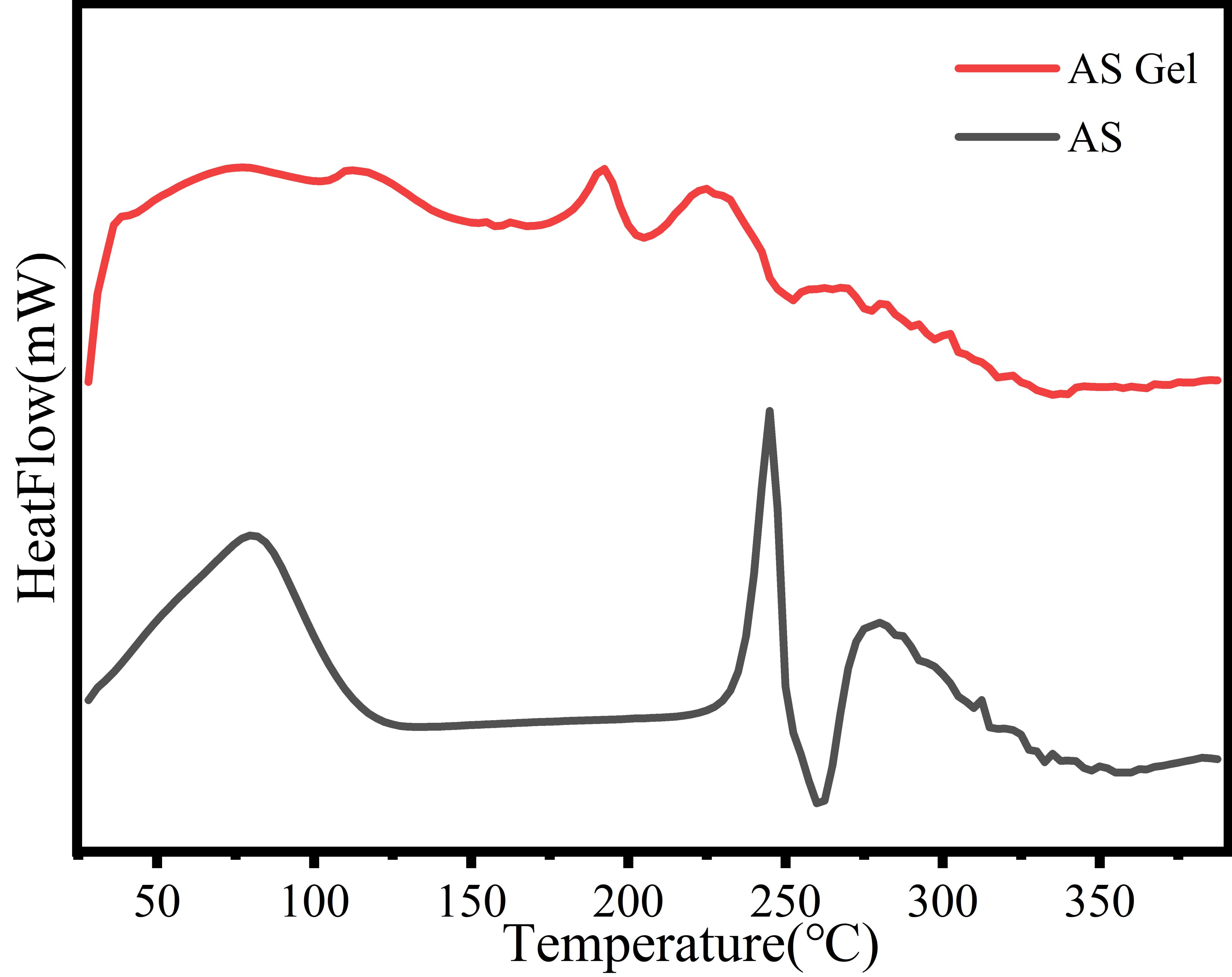

Supplement: Supplementary file 1 [file DataSheet1.zip › original image/Figure 3A.jpg]

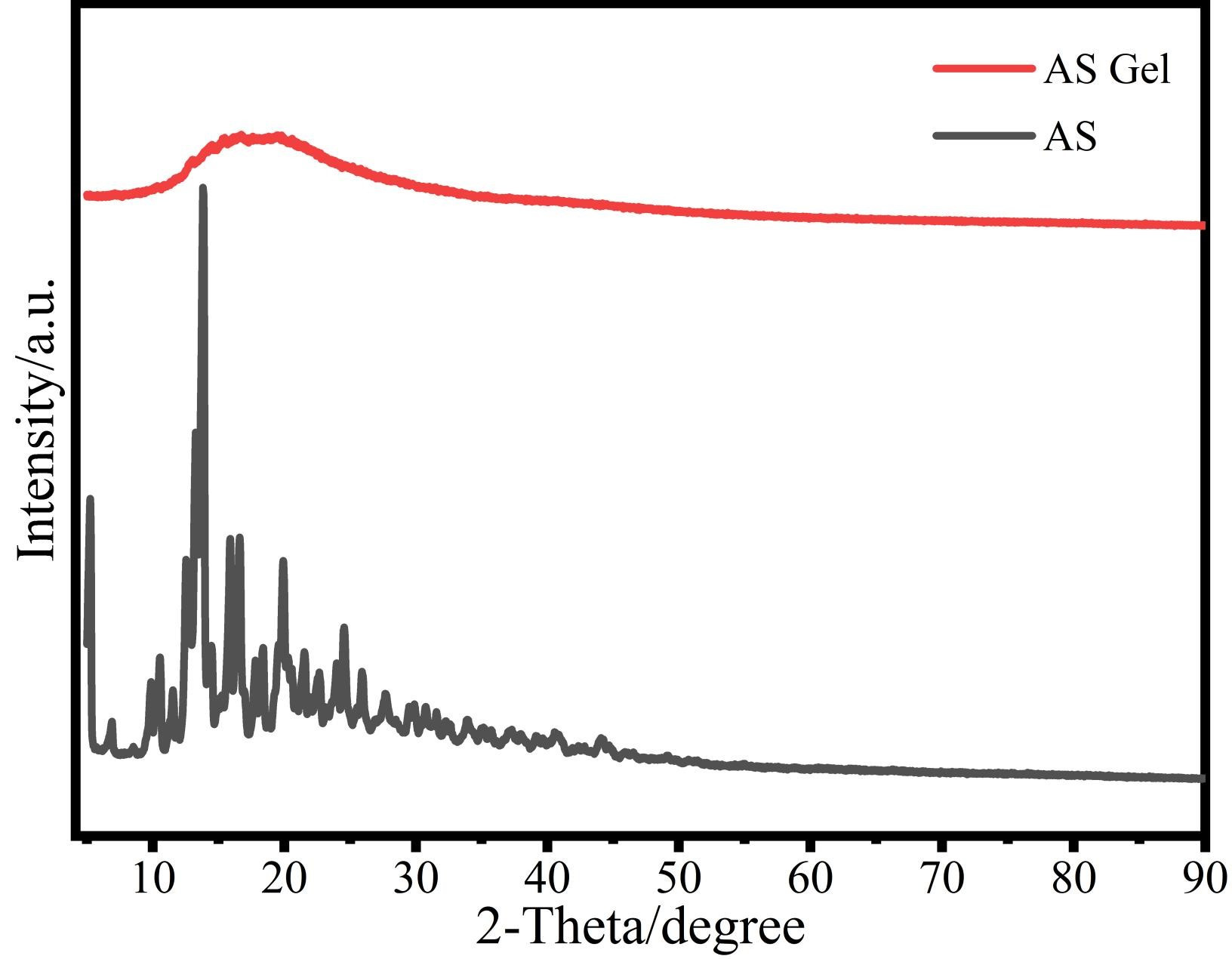

Supplement: Supplementary file 1 [file DataSheet1.zip › original image/Figure 3B.jpg]

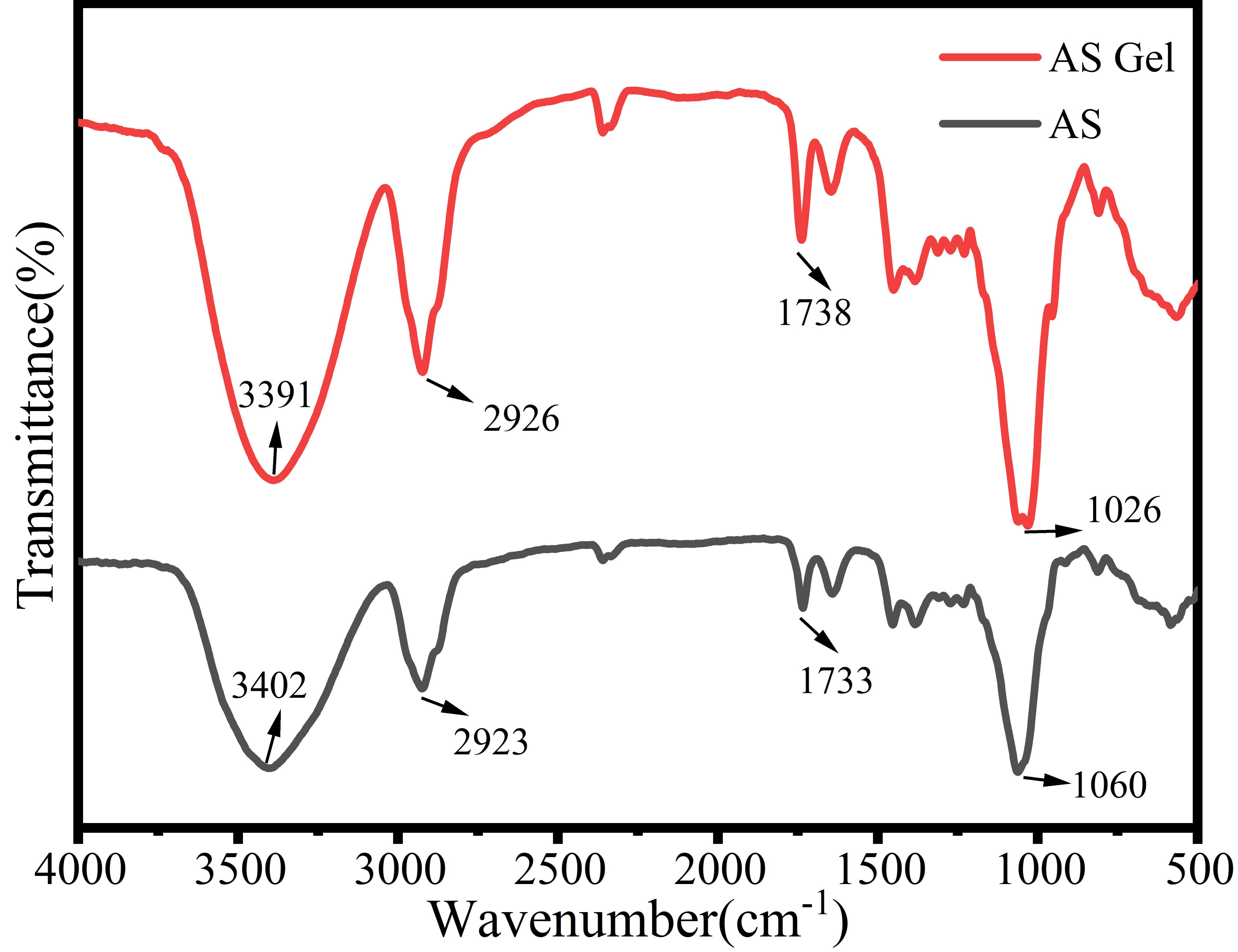

Supplement: Supplementary file 1 [file DataSheet1.zip › original image/Figure 3C.jpg]

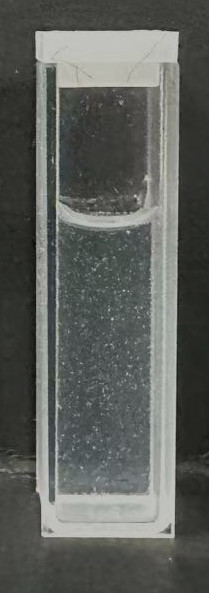

Supplement: Supplementary file 1 [file DataSheet1.zip › original image/Figure 3D-0 min.jpg]

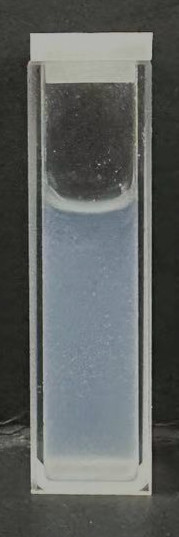

Supplement: Supplementary file 1 [file DataSheet1.zip › original image/Figure 3D-10 min.jpg]

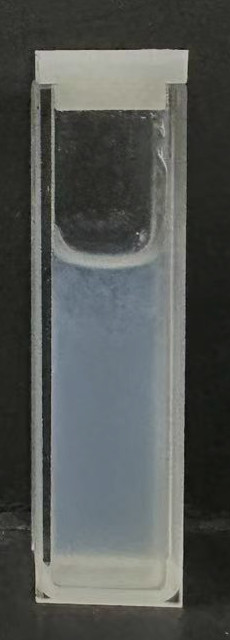

Supplement: Supplementary file 1 [file DataSheet1.zip › original image/Figure 3D-15 min-1.jpg]

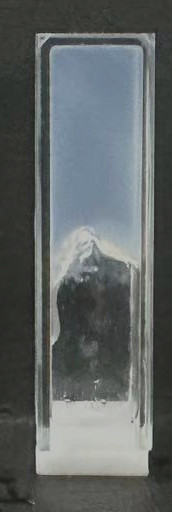

Supplement: Supplementary file 1 [file DataSheet1.zip › original image/Figure 3D-15 min-2.jpg]

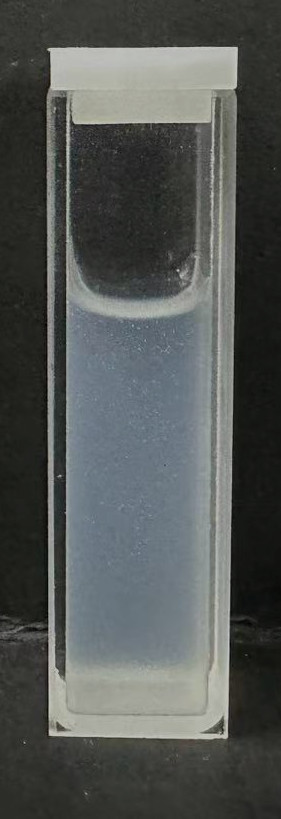

Supplement: Supplementary file 1 [file DataSheet1.zip › original image/Figure 3D-5 min.jpg]

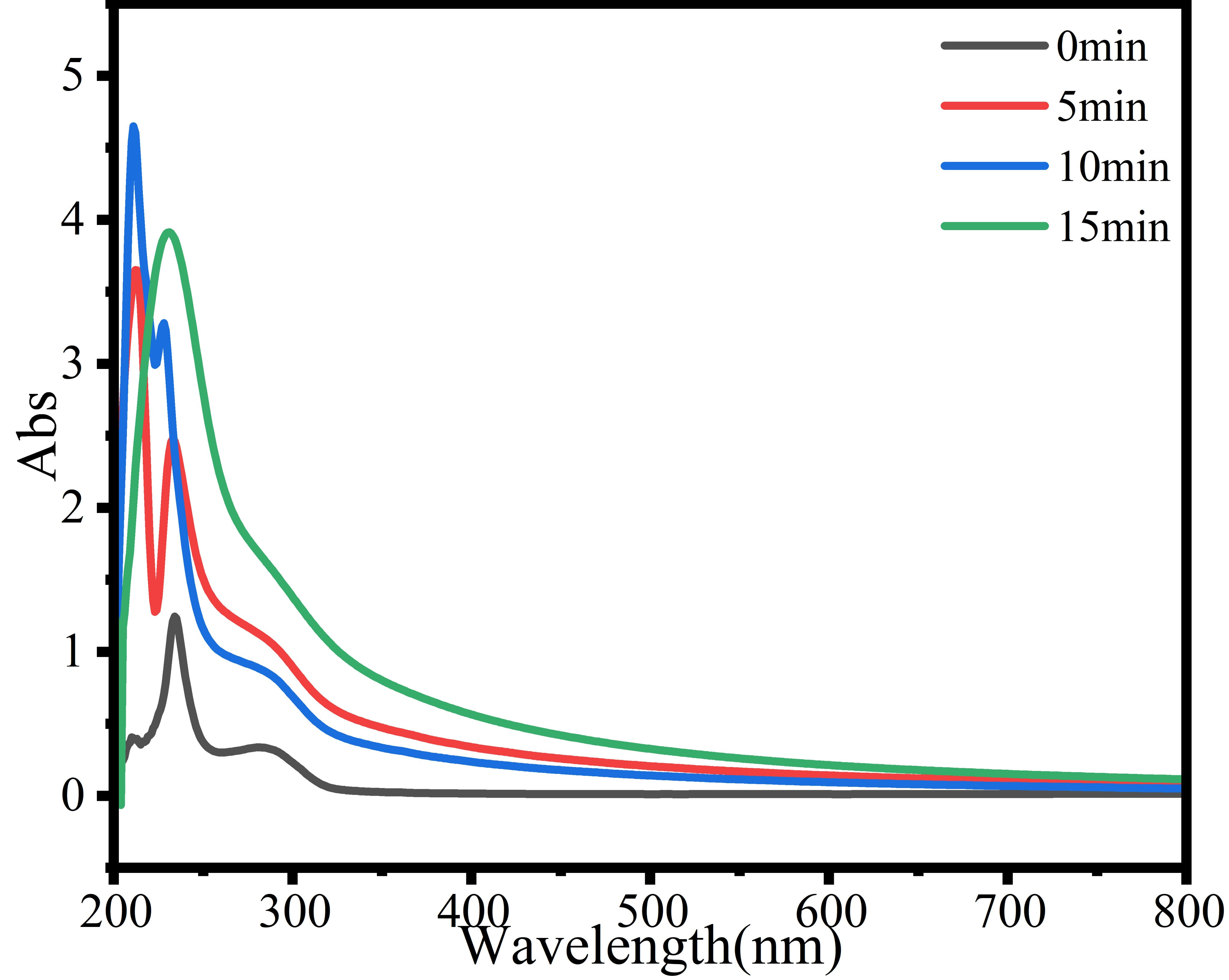

Supplement: Supplementary file 1 [file DataSheet1.zip › original image/Figure 3D.jpg]

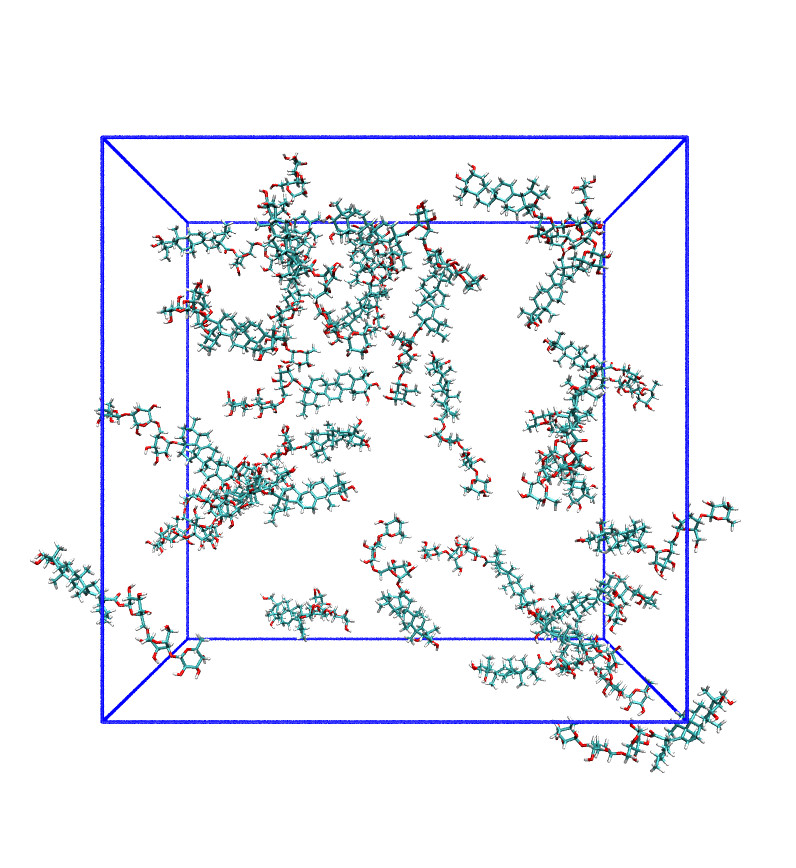

Supplement: Supplementary file 1 [file DataSheet1.zip › original image/Figure 4A-0ns.jpg]

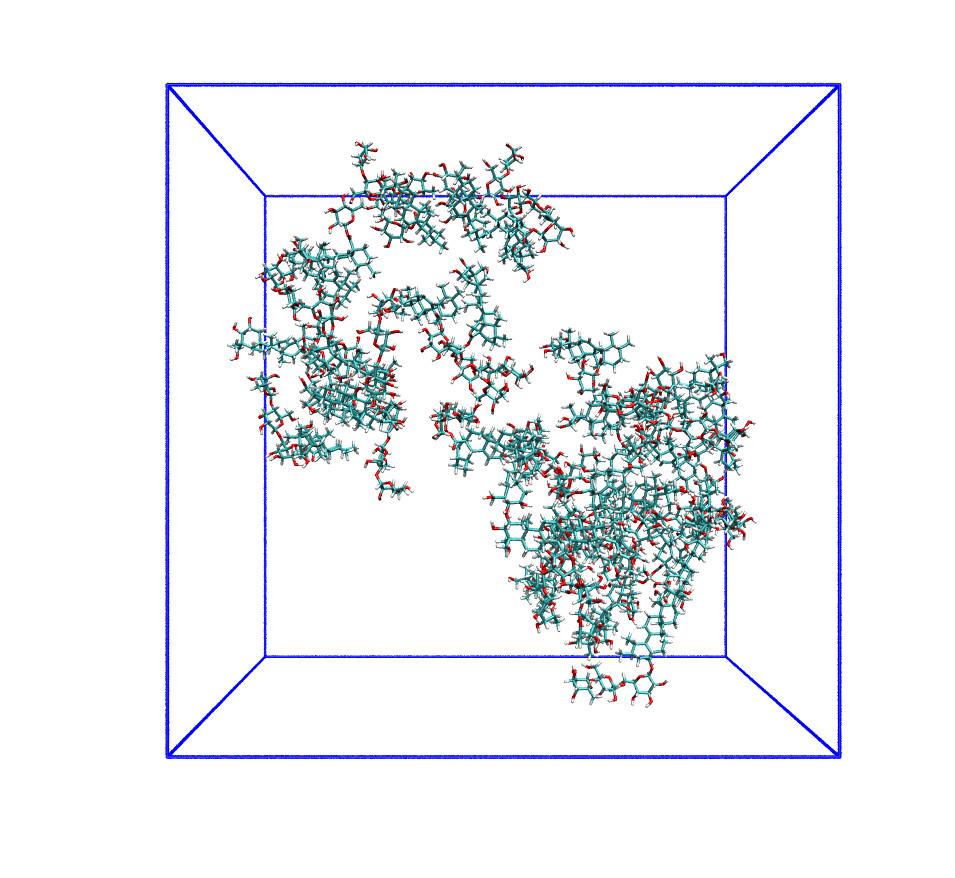

Supplement: Supplementary file 1 [file DataSheet1.zip › original image/Figure 4A-100ns.jpg]

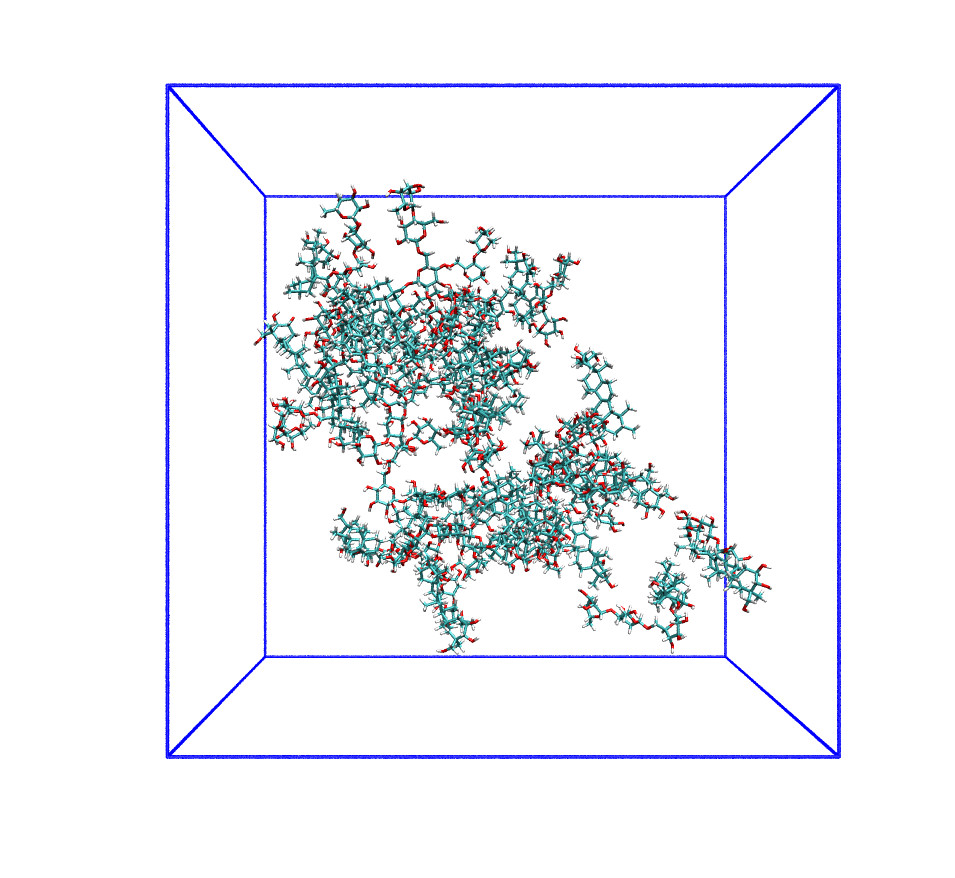

Supplement: Supplementary file 1 [file DataSheet1.zip › original image/Figure 4A-150ns.jpg]

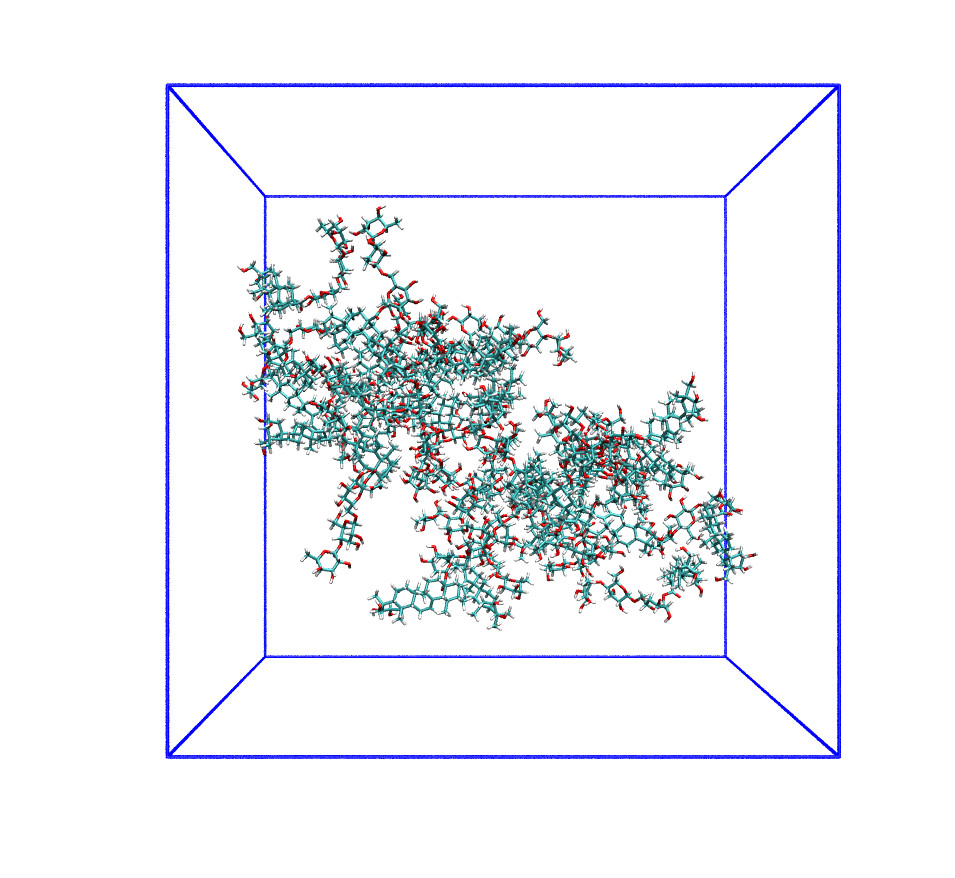

Supplement: Supplementary file 1 [file DataSheet1.zip › original image/Figure 4A-200ns.jpg]

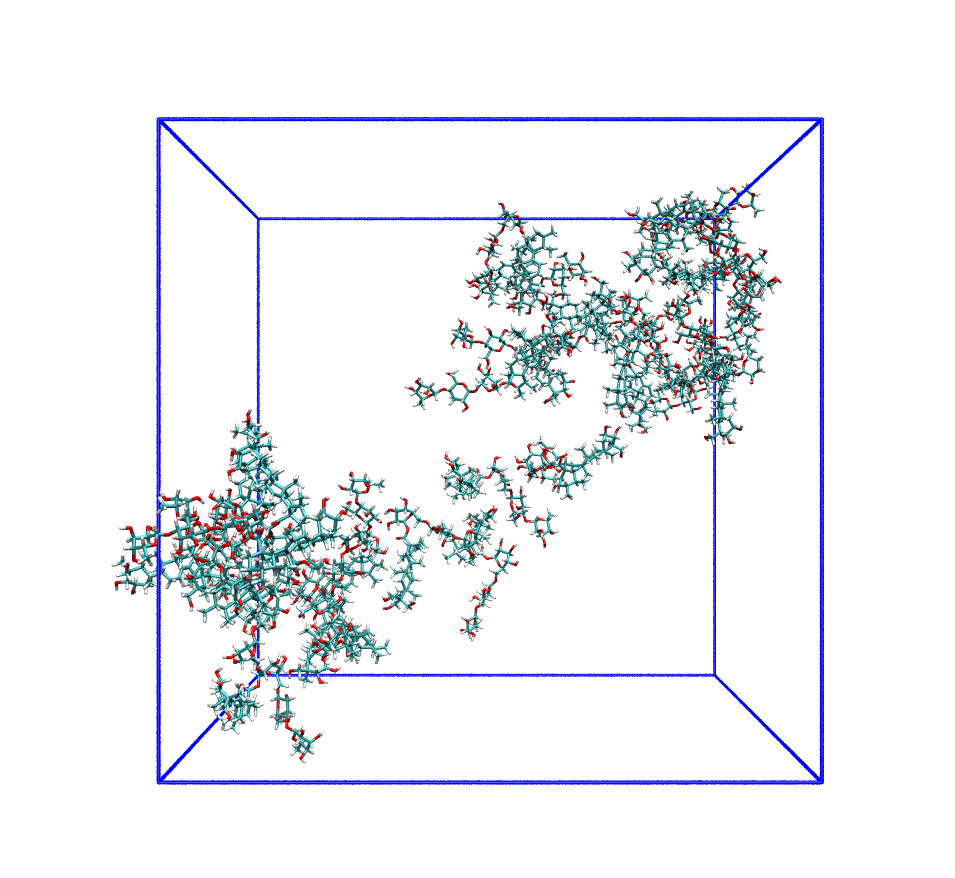

Supplement: Supplementary file 1 [file DataSheet1.zip › original image/Figure 4A-30ns.jpg]

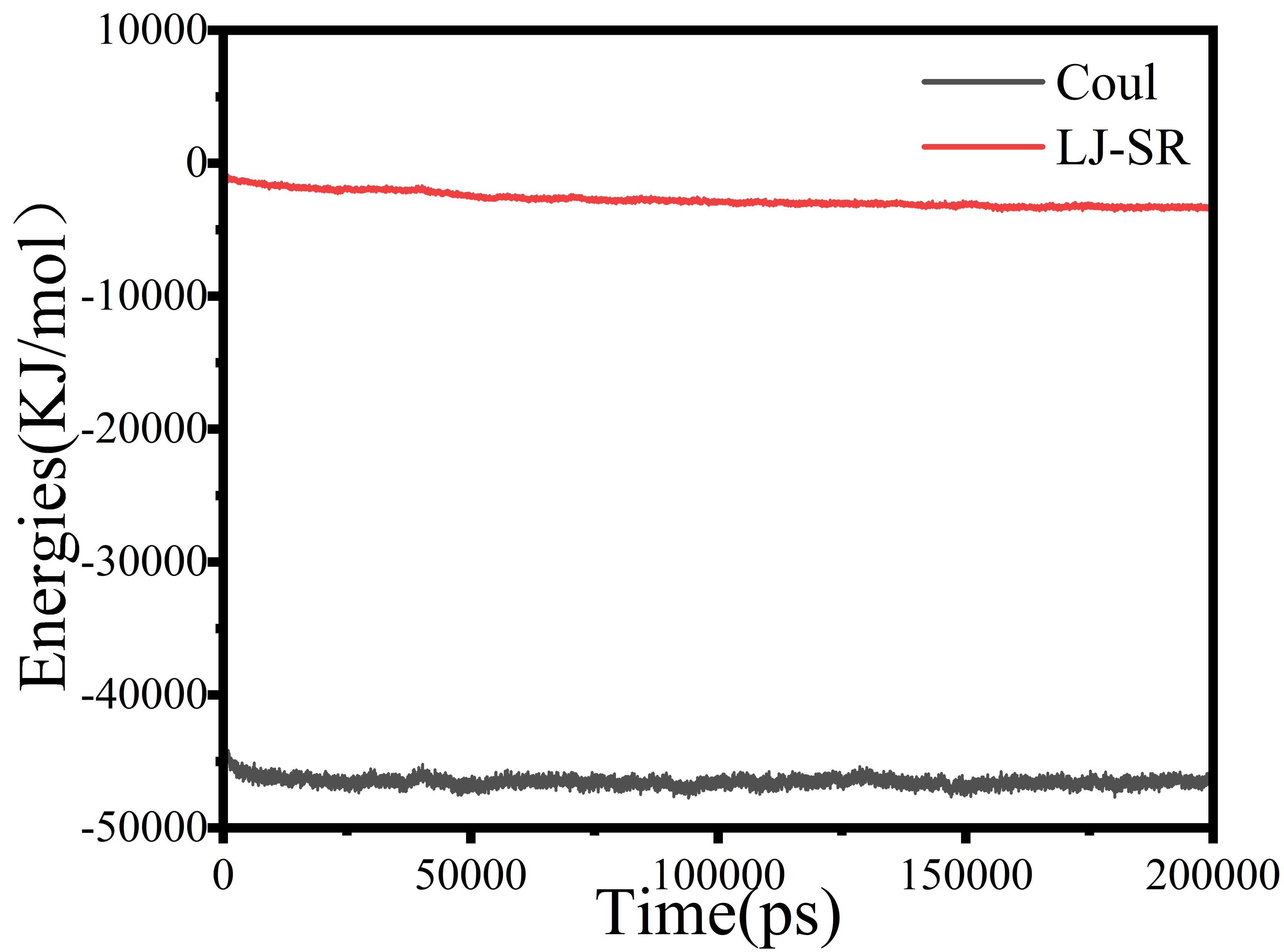

Supplement: Supplementary file 1 [file DataSheet1.zip › original image/Figure 4B.jpg]

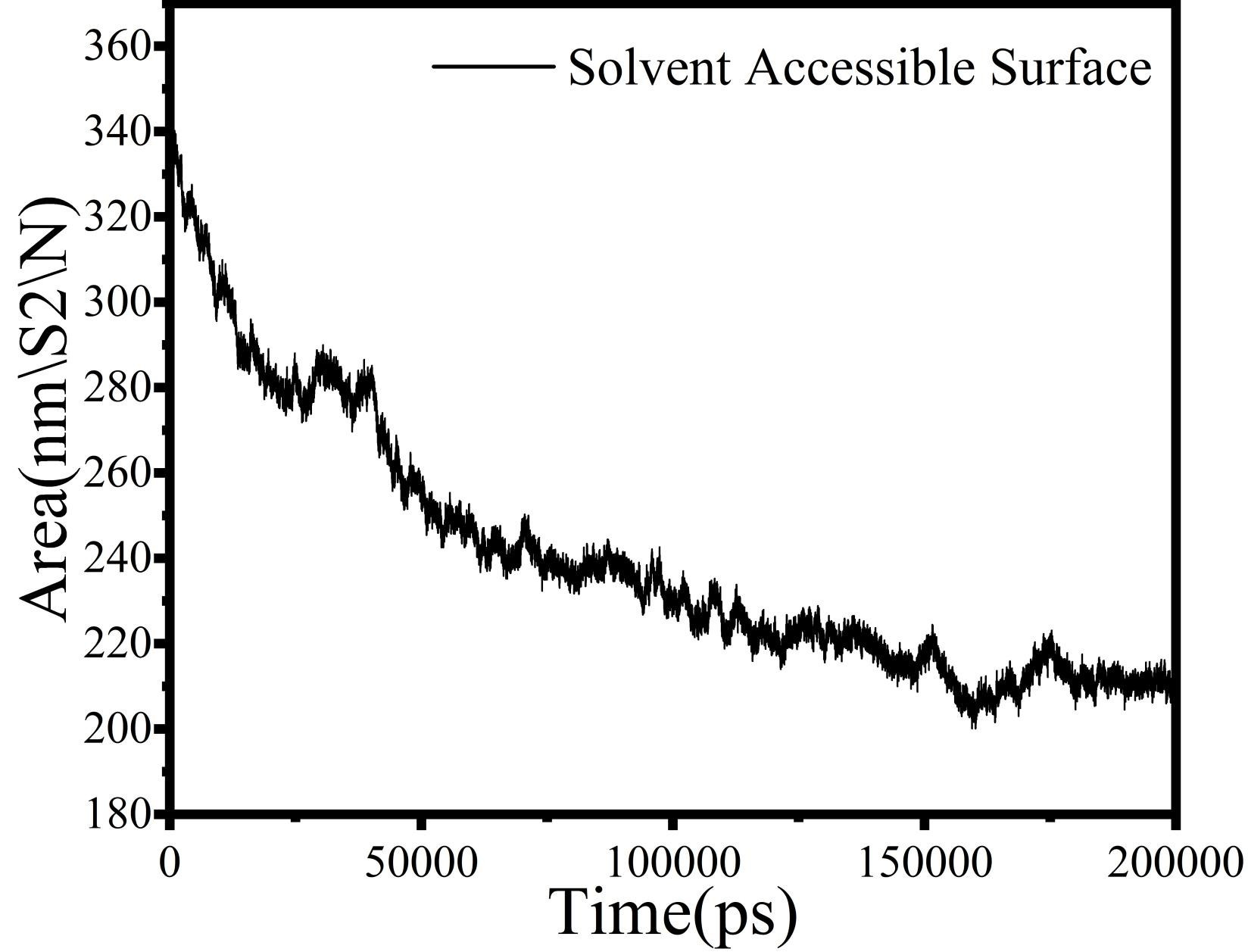

Supplement: Supplementary file 1 [file DataSheet1.zip › original image/Figure 4C.jpg]

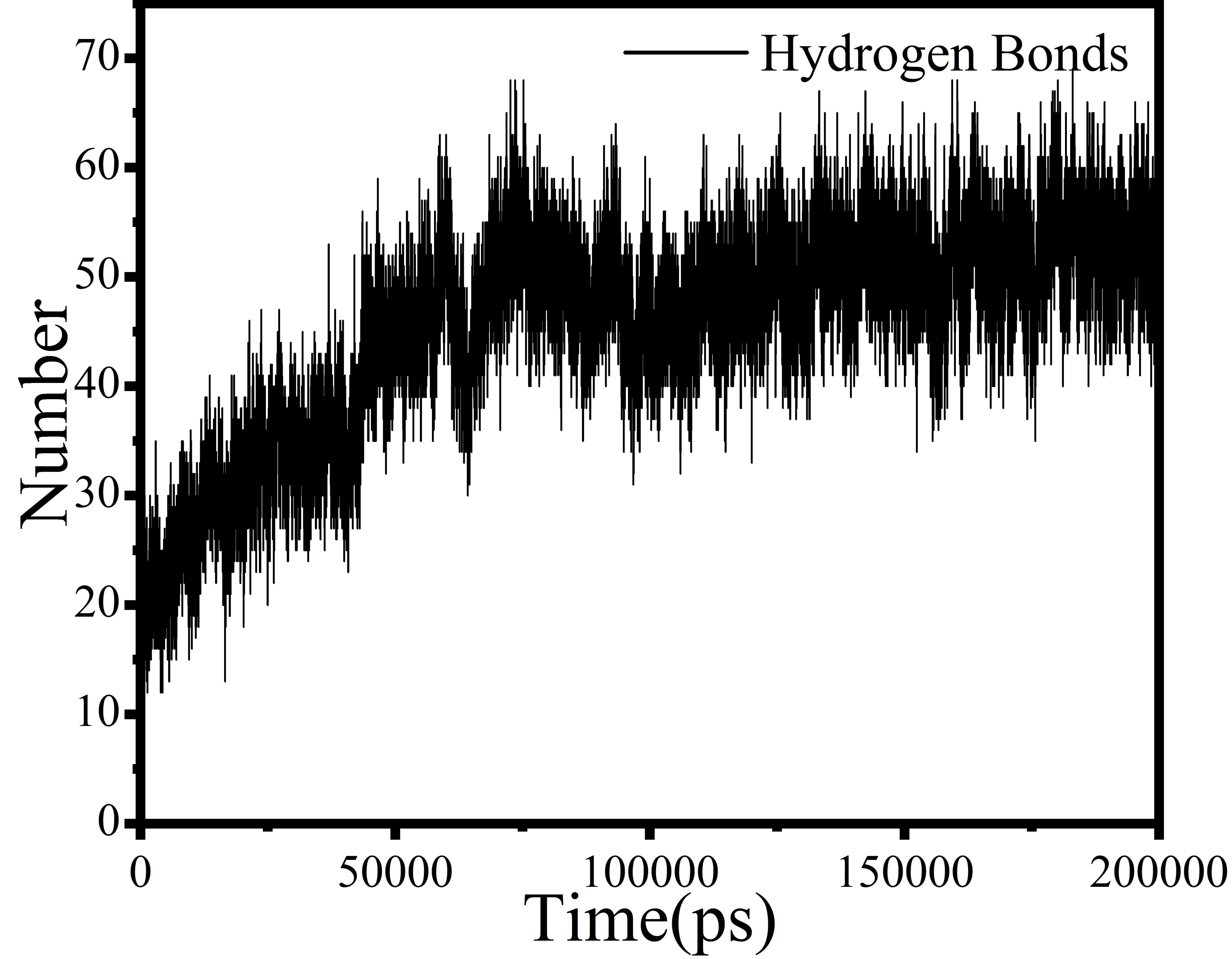

Supplement: Supplementary file 1 [file DataSheet1.zip › original image/Figure 4D.jpg]

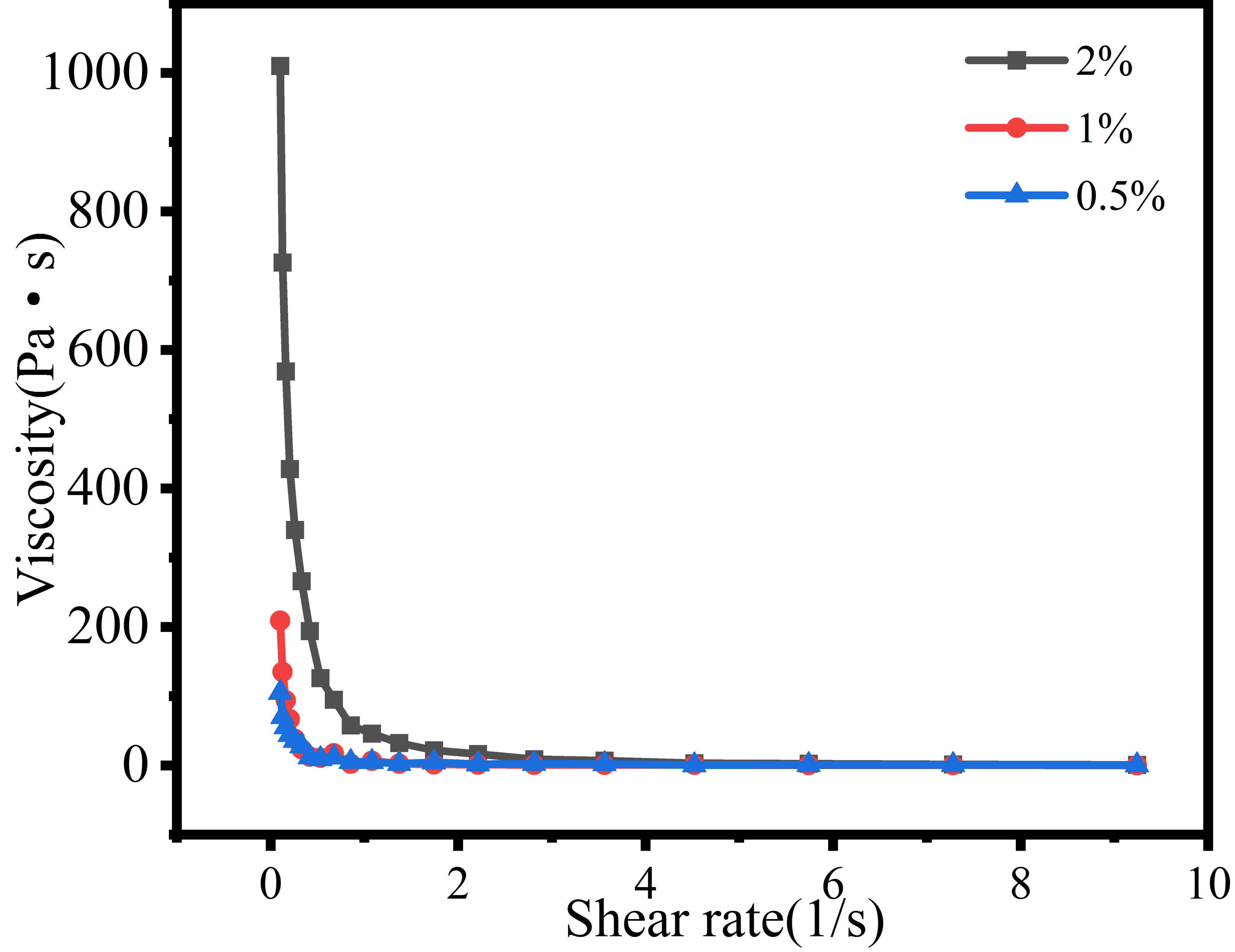

Supplement: Supplementary file 1 [file DataSheet1.zip › original image/Figure 5A.jpg]

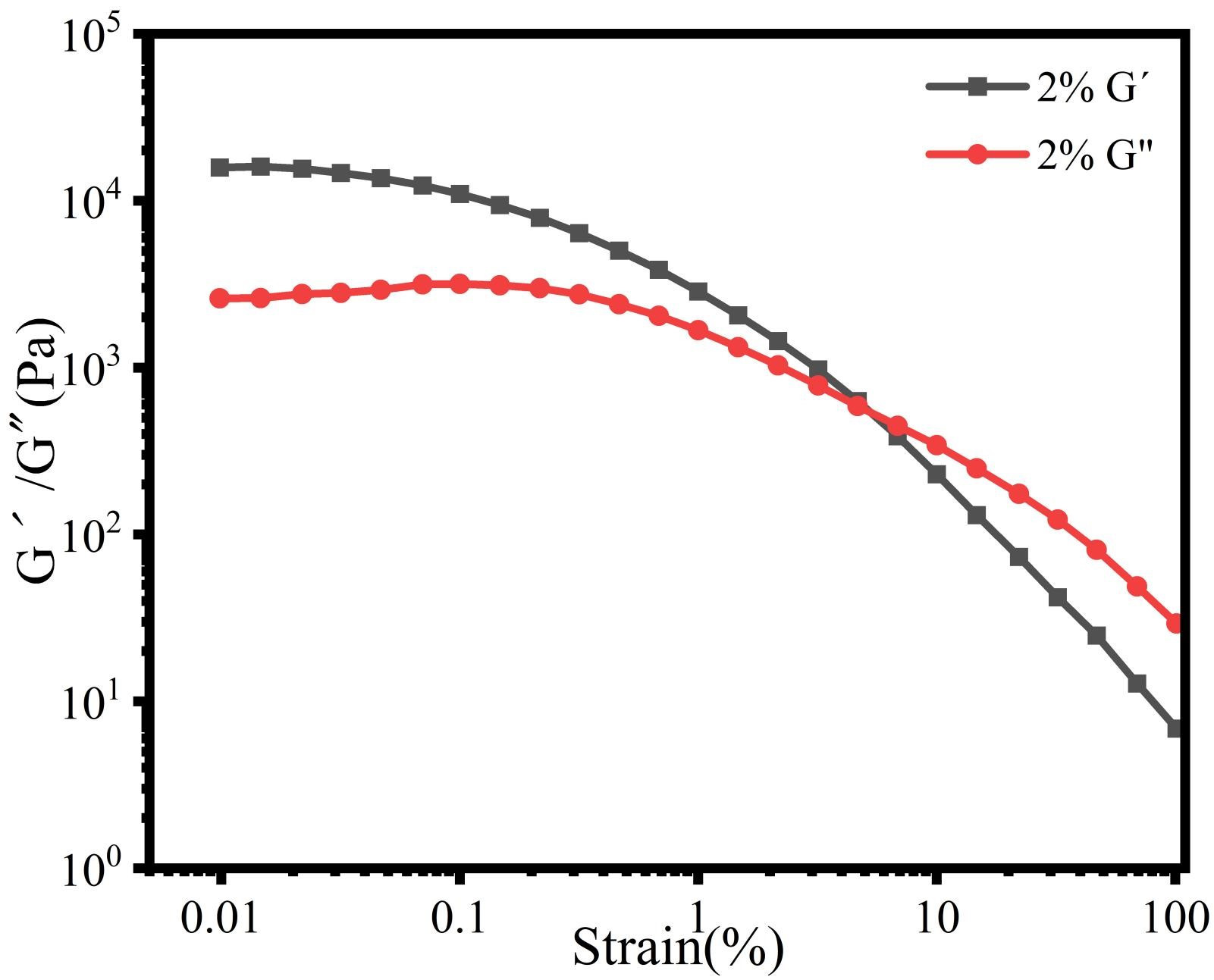

Supplement: Supplementary file 1 [file DataSheet1.zip › original image/Figure 5B.jpg]

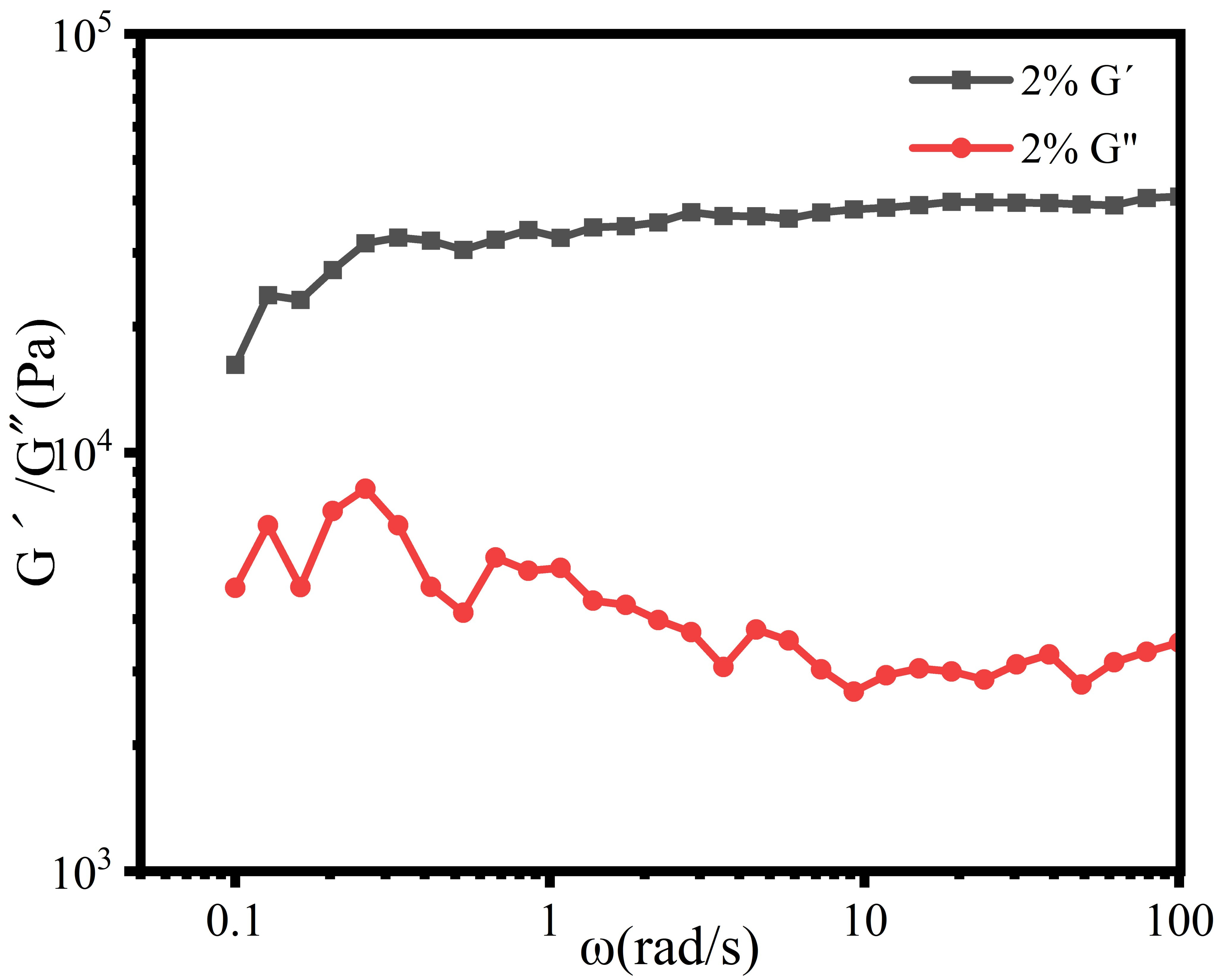

Supplement: Supplementary file 1 [file DataSheet1.zip › original image/Figure 5C.jpg]

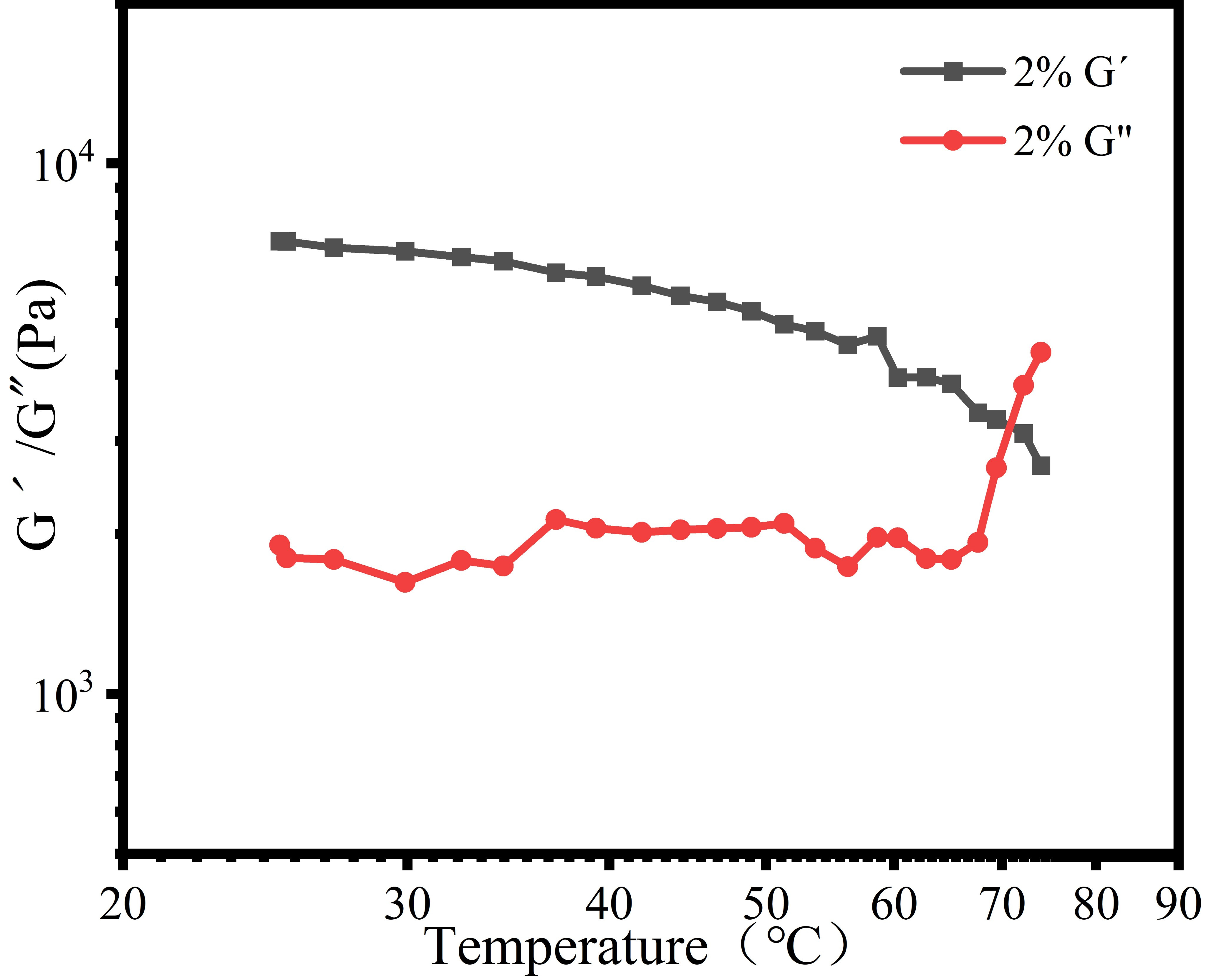

Supplement: Supplementary file 1 [file DataSheet1.zip › original image/Figure 5D.jpg]

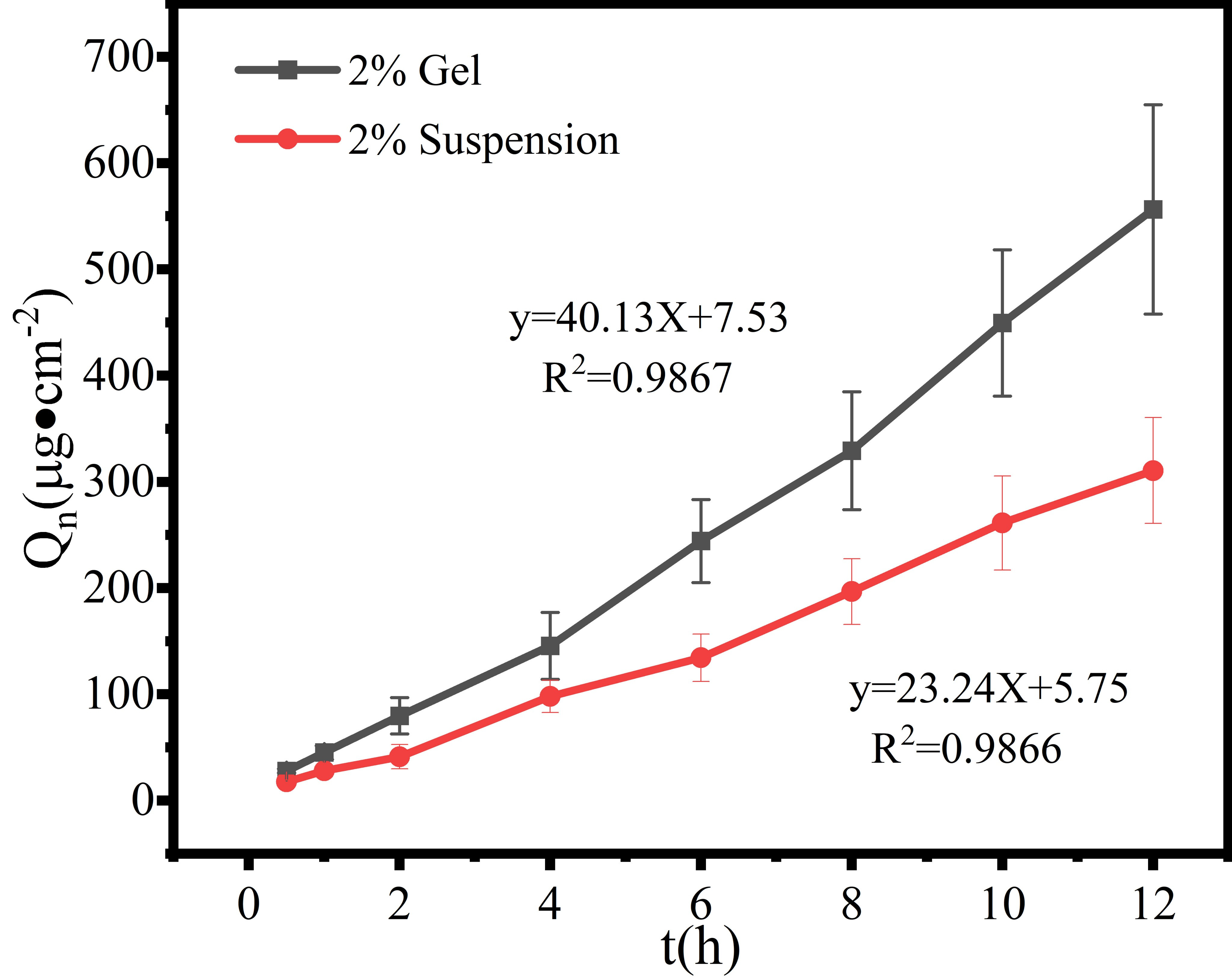

Supplement: Supplementary file 1 [file DataSheet1.zip › original image/Figure 6A.jpg]

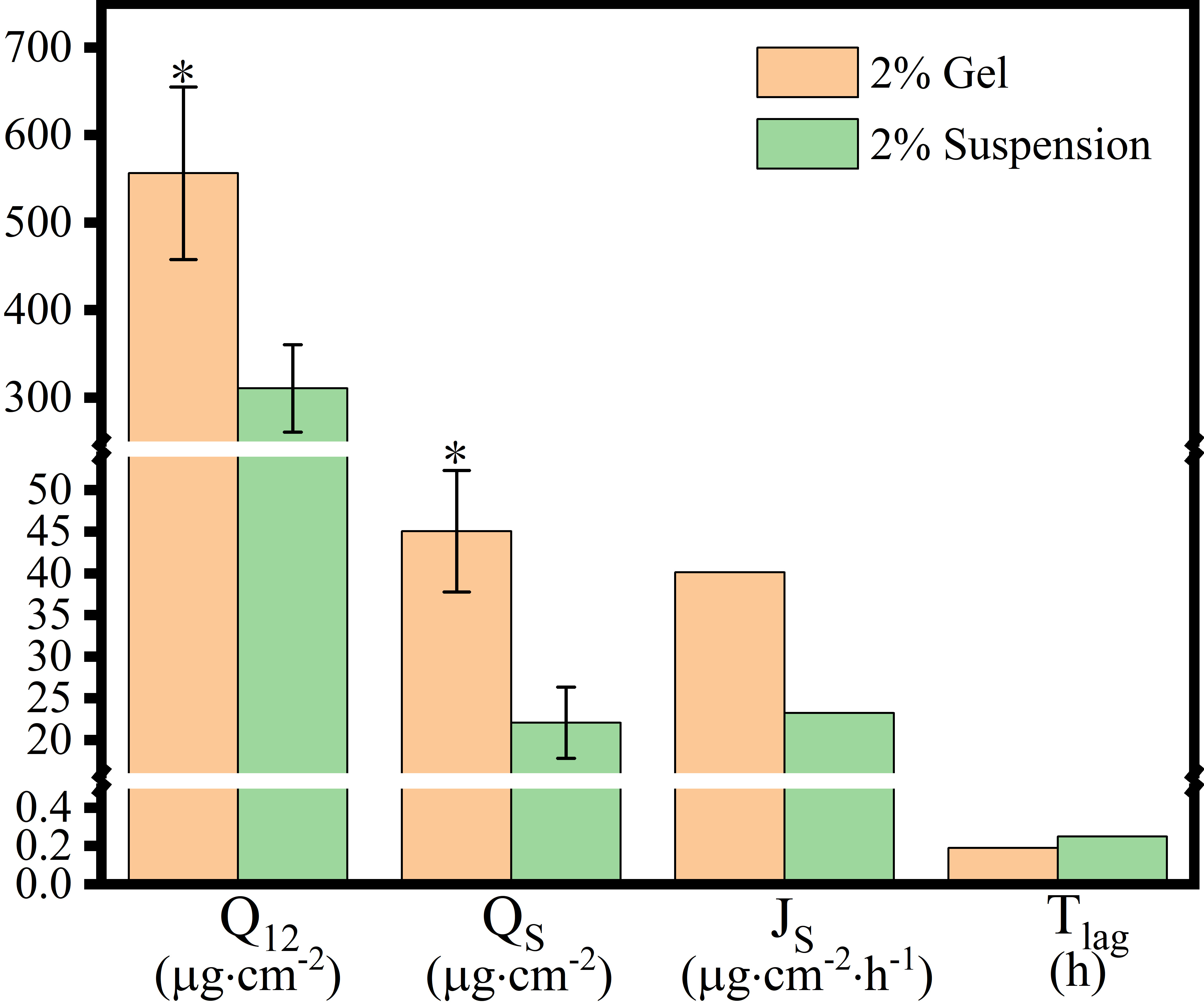

Supplement: Supplementary file 1 [file DataSheet1.zip › original image/Figure 6B.jpg]

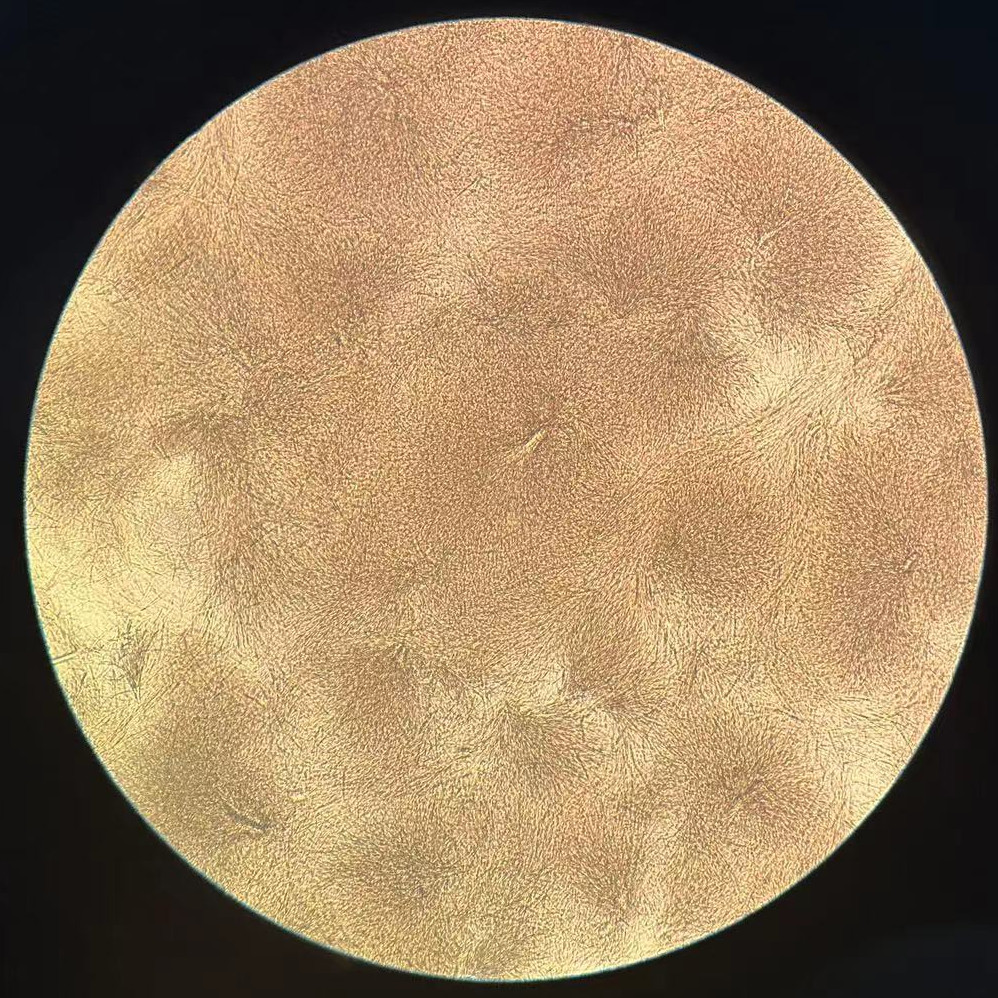

Supplement: Supplementary file 1 [file DataSheet1.zip › original image/Figure 7A-0 d (microscopy).jpg]

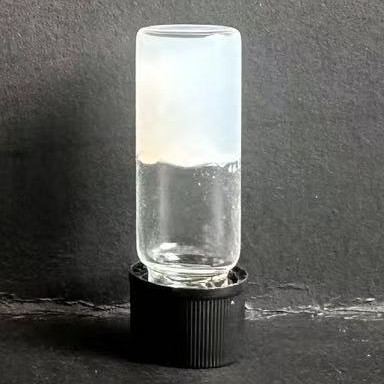

Supplement: Supplementary file 1 [file DataSheet1.zip › original image/Figure 7A-0 d.jpg]

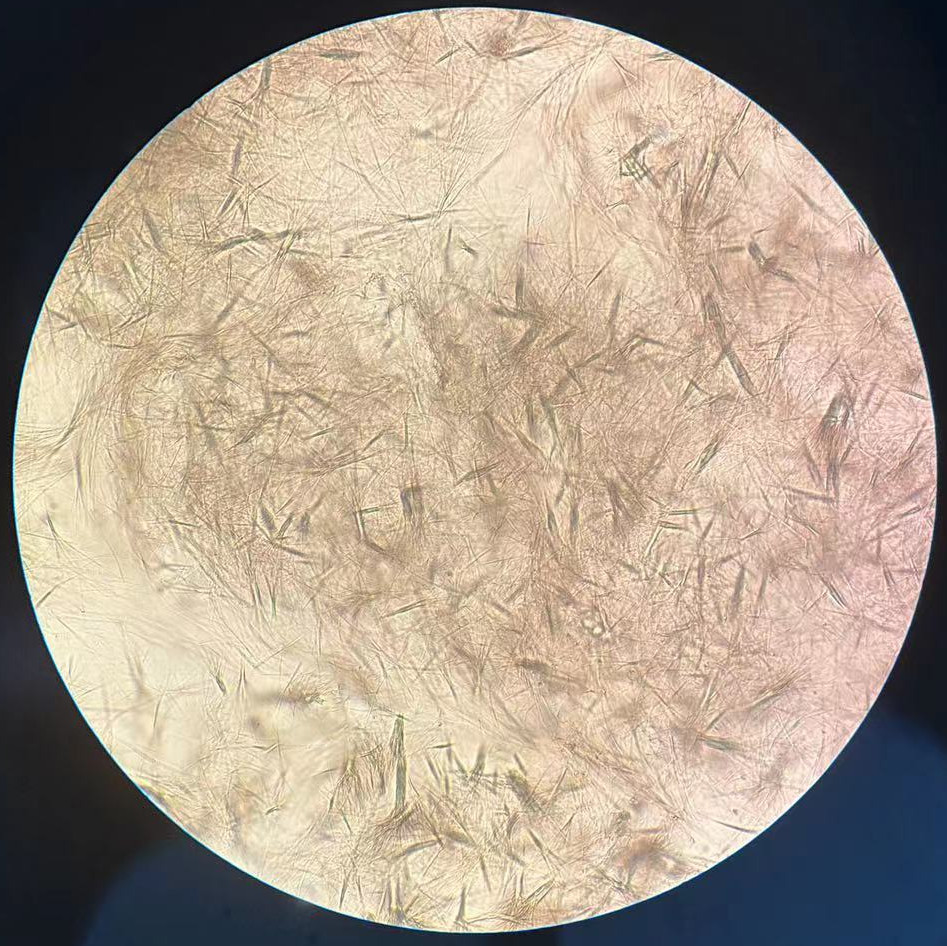

Supplement: Supplementary file 1 [file DataSheet1.zip › original image/Figure 7A-30 d (microscopy).jpg]

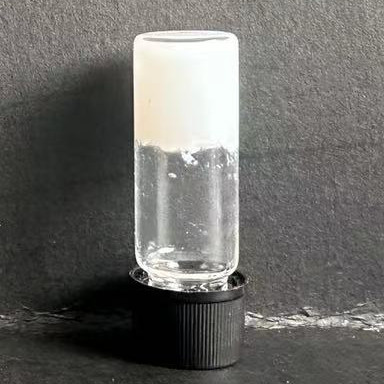

Supplement: Supplementary file 1 [file DataSheet1.zip › original image/Figure 7A-30 d.jpg]

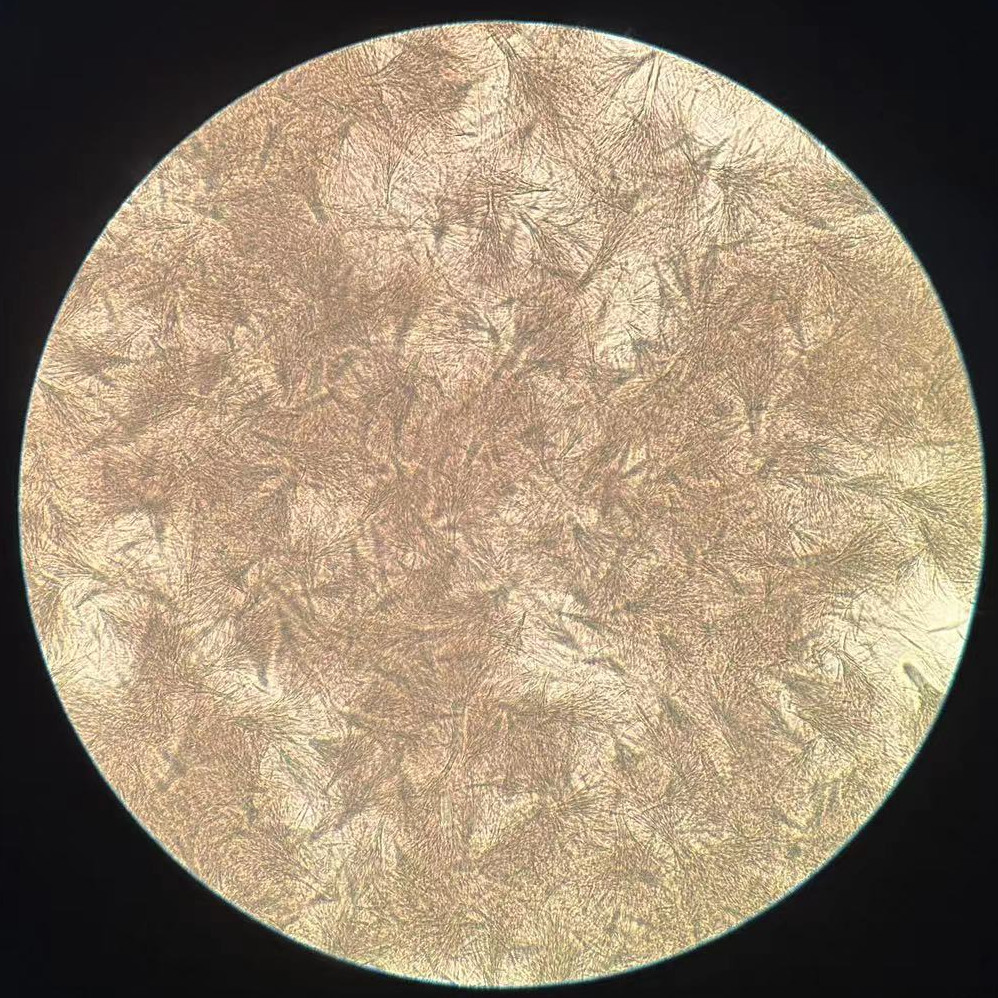

Supplement: Supplementary file 1 [file DataSheet1.zip › original image/Figure 7A-60 d (microscopy).jpg]

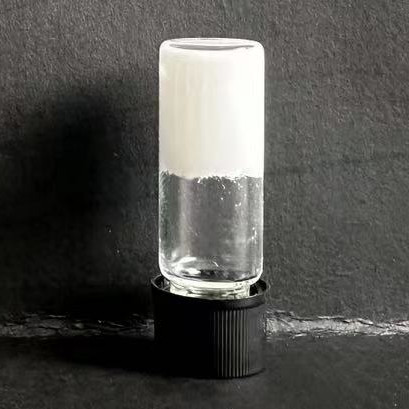

Supplement: Supplementary file 1 [file DataSheet1.zip › original image/Figure 7A-60 d.jpg]

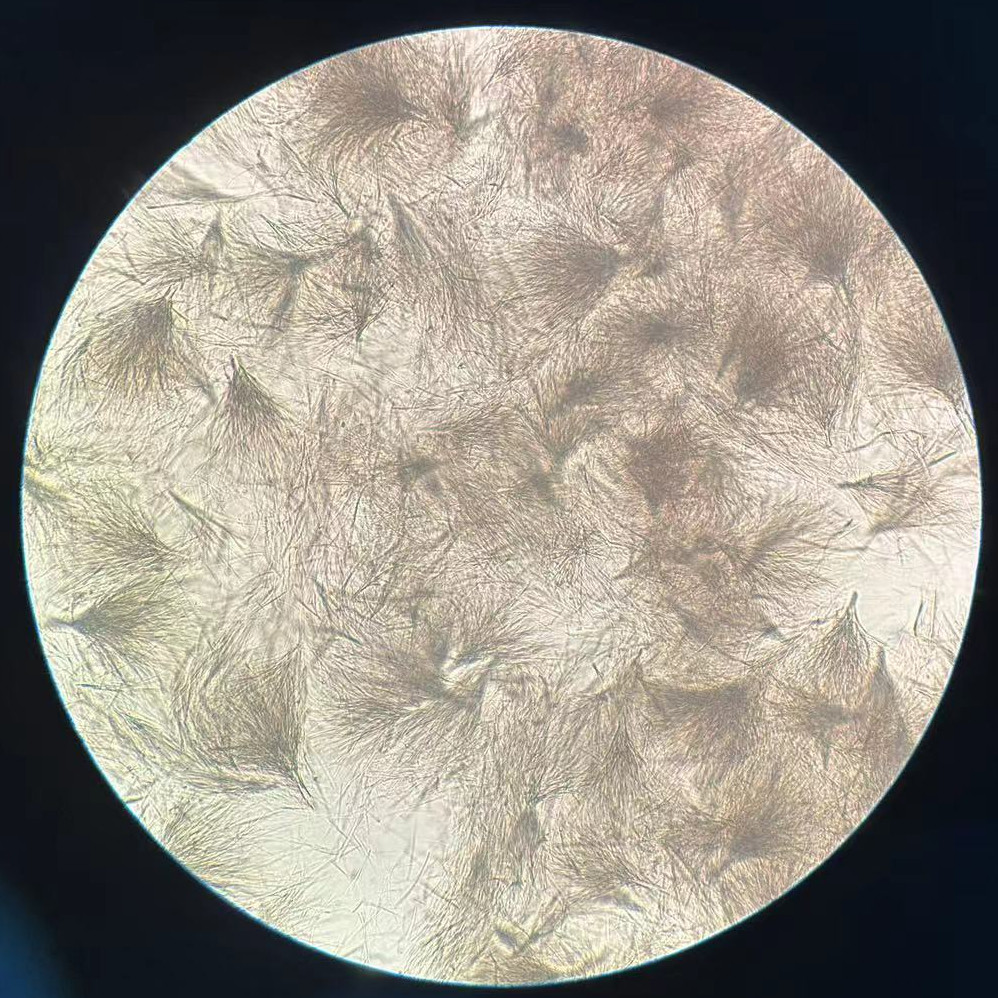

Supplement: Supplementary file 1 [file DataSheet1.zip › original image/Figure 7A-90 d (microscopy).jpg]

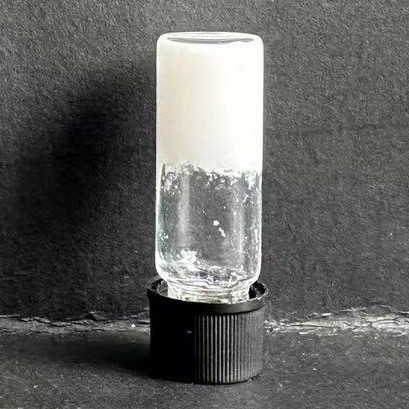

Supplement: Supplementary file 1 [file DataSheet1.zip › original image/Figure 7A-90 d.jpg]

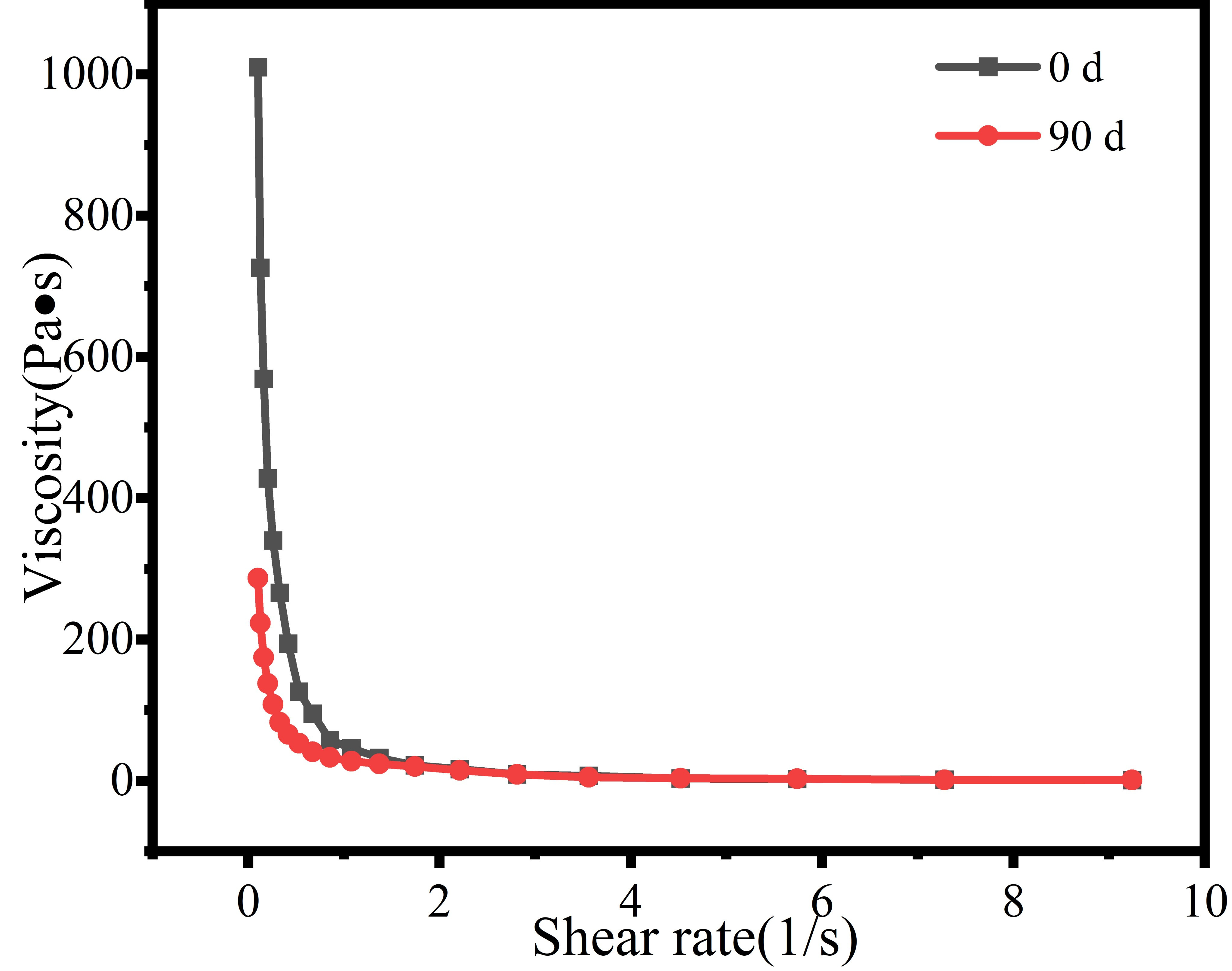

Supplement: Supplementary file 1 [file DataSheet1.zip › original image/Figure 7B.jpg]
